# Plant microbiota feedbacks through dose-responsive expression of general non-self response genes

**DOI:** 10.1038/s41477-024-01856-z

**Published:** 2024-12-03

**Authors:** Andreas Keppler, Michelle Roulier, Sebastian Pfeilmeier, Gabriella C. Petti, Anna Sintsova, Benjamin A. Maier, Miriam Bortfeld-Miller, Shinichi Sunagawa, Cyril Zipfel, Julia A. Vorholt

**Affiliations:** 1 Institute of Microbiology, ETH Zurich, Zurich, Switzerland; 2 Institute of Plant and Microbial Biology, Zurich-Basel Plant Science Center, University of Zurich, Zurich, Switzerland; 3 Sainsbury Laboratory, University of East Anglia, Norwich Research Park, Norwich, UK

**Keywords:** Microbe, Microbiology

## Abstract

The ability of plants to perceive and react to biotic and abiotic stresses is critical for their health. We recently identified a core set of genes consistently induced by members of the leaf microbiota, termed general non-self response (GNSR) genes. Here we show that GNSR components conversely impact leaf microbiota composition. Specific strains that benefited from this altered assembly triggered strong plant responses, suggesting that the GNSR is a dynamic system that modulates colonization by certain strains. Examination of the GNSR to live and inactivated bacteria revealed that bacterial abundance, cellular composition and exposure time collectively determine the extent of the host response. We link the GNSR to pattern-triggered immunity, as diverse microbe- or danger-associated molecular patterns cause dynamic GNSR gene expression. Our findings suggest that the GNSR is the result of a dose-responsive perception and signalling system that feeds back to the leaf microbiota and contributes to the intricate balance of plant–microbiome interactions.

## Abstract (web summary)

The plant general non-self response system is triggered by leaf microbiota members and, in turn, impacts their colonization.

## Main

Plants live in a close and dynamic association with their microbiota, a diverse consortium of microorganisms of which bacteria are the most abundant members. The plant microbiota assembles in a similar manner across healthy plants and is crucial in mitigating various abiotic and biotic stresses, as well as promoting plant growth and development^1,2^. The microbiota contributes to plant protection through direct mechanisms of microbe–microbe interactions but also indirectly via the plant^3–6^. Such indirect protection suggests that plants perceive the microbiota or specific members. Consistently, there is increasing evidence that the plant innate immune system contributes to the shaping and maintenance of this homeostatic state^7–13^.

Plants deploy pattern-recognition receptors (PRRs) to detect and respond to potentially pathogenic microorganisms by binding microbe-associated molecular patterns (MAMPs) or plant-derived danger-associated molecular patterns (DAMPs)^14,15^. Perception of these non-self or perturbed-self signals, respectively, is propagated by intricate signalling networks and results in pattern-triggered immunity (PTI), a first line of inducible defence that microorganisms need to overcome to colonize plants^16,17^. While PTI has been extensively investigated in the context of pathogens, MAMPs are conserved across pathogenic and non-pathogenic microorganisms^2^. In fact, pathogens and commensals can elicit overlapping responses in plants^3^, and some commensals can evade recognition by the plant immune system, in resemblance to pathogens^18–20^. This provokes the question of how plants regulate their responses to different colonizers, particularly in the presence of a complex microbiota representing a plethora of diverse perception signals.

In a systematic study characterizing transcriptional and metabolic responses in *Arabidopsis thaliana*, we recently identified a molecular response that is consistently elicited by leaf microbiota strains upon colonization^21^. This response is led by a set of genes, termed general non-self response (GNSR) genes, which are indicative of the extent to which the host transcriptome is reprogrammed in response to bacterial colonization of leaves and comprise several genes previously implicated in plant immunity^21–26^. Indeed, the infection of plants associated with a natural microbiota resulted in increased abundances of the foliar pathogen *Pseudomonas syringae* pv. *tomato* DC3000 (Pst) in mutants lacking individual GNSR components^21^. Notable among them was *CYP71A12*, which encodes a cytochrome P450 monooxygenase that contributes to the synthesis of defence-associated tryptophan derivatives and is upregulated in response to pathogen encounter^26^ and beneficial microorganisms in induced systemic resistance^5,25^. *CYP71A12* exhibited the strongest induction among all plant genes under different bacterial treatment conditions, showed the highest dynamic range in expression, was the best predictor of overall plant transcriptional reprogramming (*R*^2^ = 0.89) and is required for effective resistance against pathogens^5,21,26^, highlighting its central role in response to non-self perception. Intriguingly, the intensity of the overall host response and that of the GNSR both correlated significantly with bacterial population size, suggesting a relationship between the exposure to leaf microbiota strains and plant response intensity^21^. In line with this, a recent study found that plant immune responses can be induced by non-pathogenic bacteria when inoculated with high densities exceeding their inherent colonization ability^27^. Together, this suggests that plants monitor bacterial abundance to regulate transcriptional adaptations to their leaf microbiota. The dynamic adaptability of plants to the microbiota is further supported by recent work in which the development of plant immunocompetence has been examined and microbiota-induced GNSR genes have been suggested to contribute to PTI^28^, underscoring their role as a fundamental component of the immune system and plant–microbiota interactions.

Here we show that the microbiota-responsive GNSR genes impact colonization by leaf microbiota members. We then investigate the immunomodulatory capability of members of the leaf microbiota, demonstrating both an immunostimulatory and a suppressive effect on GNSR gene induction, and show the temporal dynamics of the response. We reveal that the GNSR is responsive to microbiota abundance and its MAMPs in a dose-dependent manner, and we link the GNSR to classical PTI transcriptional reprogramming^29^, unifying these previously disparately described responses. The inclusion of the GNSR within PTI is additionally supported by in silico analyses that reveal a common regulatory interaction network orchestrating non-self recognition. We further highlight that our current understanding of signalling pathways is still incomplete, opening intriguing perspectives on the upstream signalling events of microbiota perception that converge to a centralized plant immune response.

## Results

### GNSR components impact leaf microbiota assembly

**Extended Data Fig. 1:**
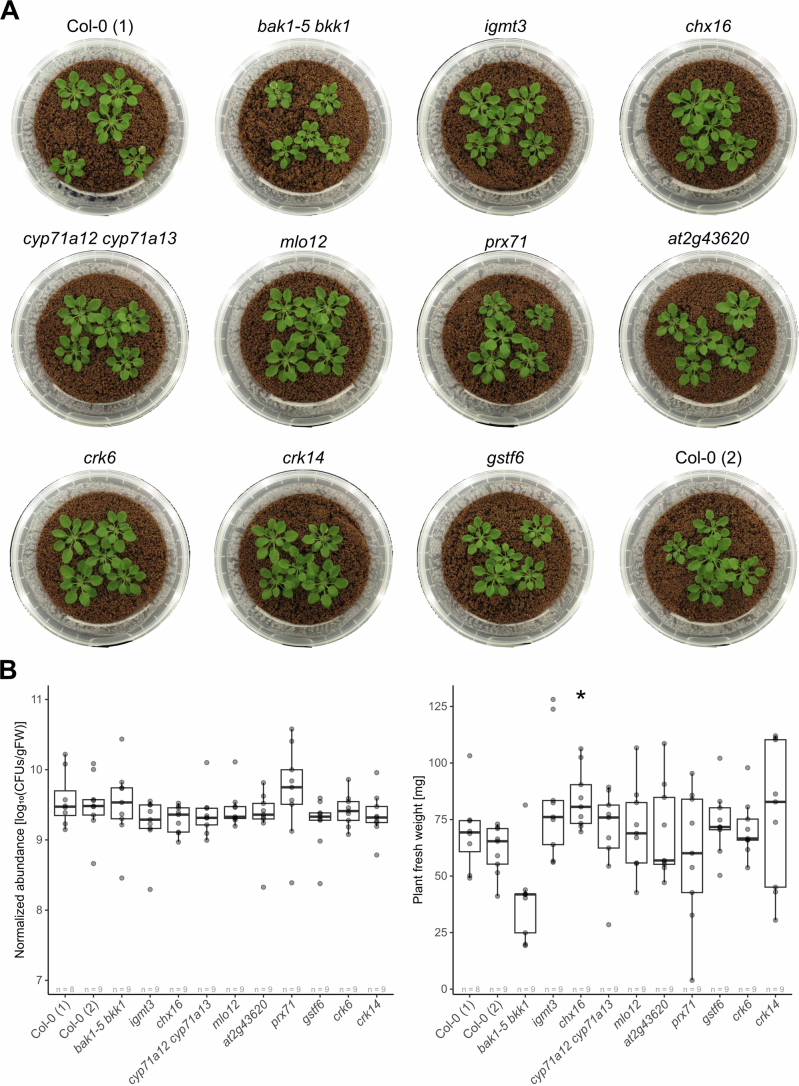
Plant phenotypes of microbiota profiling. (**A**) Representative images of *A. thaliana* Col-0 wild-type, *bak1-5 bkk1*, and GNSR mutant plants (indicated above each picture) inoculated with a synthetic microbiota of 137 strains (SynCom-137). Pictures taken immediately before harvest (3.5 weeks after inoculation). Data from one of five independent experiments, where similar phenotypes were observed. (**B**) Overall colonization levels of the synthetic microbiota SynCom-137 (left) and plant fresh weight (right) (y-axis) across Col-0, *bak1-5 bkk1*, and GNSR mutants (x-axis) 3.5 weeks after inoculation. Bars indicate median, hinges indicate first and third quartile, whiskers indicate smallest/largest value within 1.5 × IQR (interquartile range). Data from one experiment with n = 8–9 plants per condition (indicated above x-axis). Statistical significance was assessed by a two-sided t-test with Bonferroni correction using “Col-0 (2)” as reference group (*: p ≤ 0.05). Source data

The discovery of the GNSR, a recently described plant response to diverse leaf microbiota strains^21^, raises the question to what extent these core response genes retroact on the microbiota by affecting its assembly. To address this, we used a synthetic community approach in a previously established gnotobiotic system^30^ and assessed leaf microbiota composition by bacterial 16S rRNA gene sequencing 3.5 weeks after inoculation of *Arabidopsis* GNSR mutant plant lines. The tested GNSR mutants harboured deficiencies in genes affecting the synthesis of secondary metabolites (*IGMT3*, *CYP71A12*, *CYP71A13* and *GSTF6*), pH and ion homeostasis (*CHX16*), stomatal immunity (*PRX71*) or other processes implicated in defence (*AT2G43620*, *CRK14*, *CRK6* and *MLO12*)^21,24,26,31–36^. Of particular interest were the mutants *igmt3*, *cyp71a12* *cyp71a13*, *gstf6*, *chx16*, *at2g43620* and *mlo12*, as they were previously shown to be more susceptible to disease upon pathogen infection^21,22,31–33,36^. The synthetic microbiota comprised a taxonomically representative set of 137 bacterial strains, amplicon sequencing variants (ASVs) of the *At-*LSPHERE^9,37^ (Supplementary Table 1). Inoculation of the microbiota onto GNSR mutants did not result in disease symptoms (Extended Data Fig. 1a), and overall colonization levels were comparable to those on wild-type plants (Extended Data Fig. 1b).

**Fig. 1:**
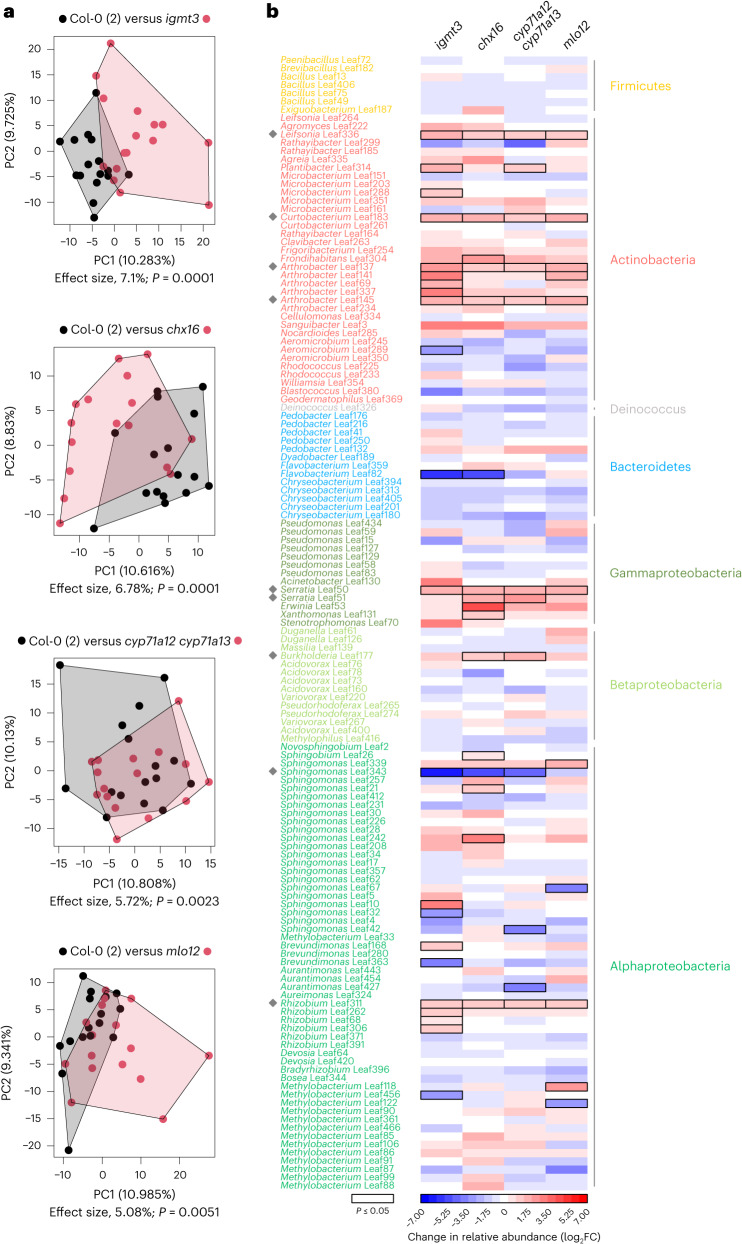
Examination of leaf microbiota composition in GNSR mutant plants. **a**, PCA of synthetic microbiota (SynCom-137) in *A. thaliana* Col-0 (black) and the indicated GNSR mutants (red) with significantly altered microbiota composition, showing overall changes in assembly. PC1 and PC2 are principal components 1 and 2, with their explained variance as indicated. Effect sizes and *P* values were calculated by PERMANOVA (10^4^ permutations) and represent the variance explained by genotype and the corresponding statistical significance, respectively (Benjamini–Hochberg adjusted). **b**, Heat map of log_2_FCs of abundance of microbiota strains in the mutant plants shown in **a** relative to the *A. thaliana* Col-0 wild type. Rectangles outlined in black indicate significant changes (*P* ≤ 0.05, two-sided Wald test, Benjamini–Hochberg adjusted). The strains are ordered according to phylogeny and coloured according to phylum or class, as indicated next to the heat map. The grey diamonds indicate consistently enriched strains, which tend to induce a strong GNSR, as shown in Extended Data Fig. 3. In **a**,**b**, the data are from one experiment with *n* = 16 plants per condition. Source data

**Extended Data Fig. 2:**
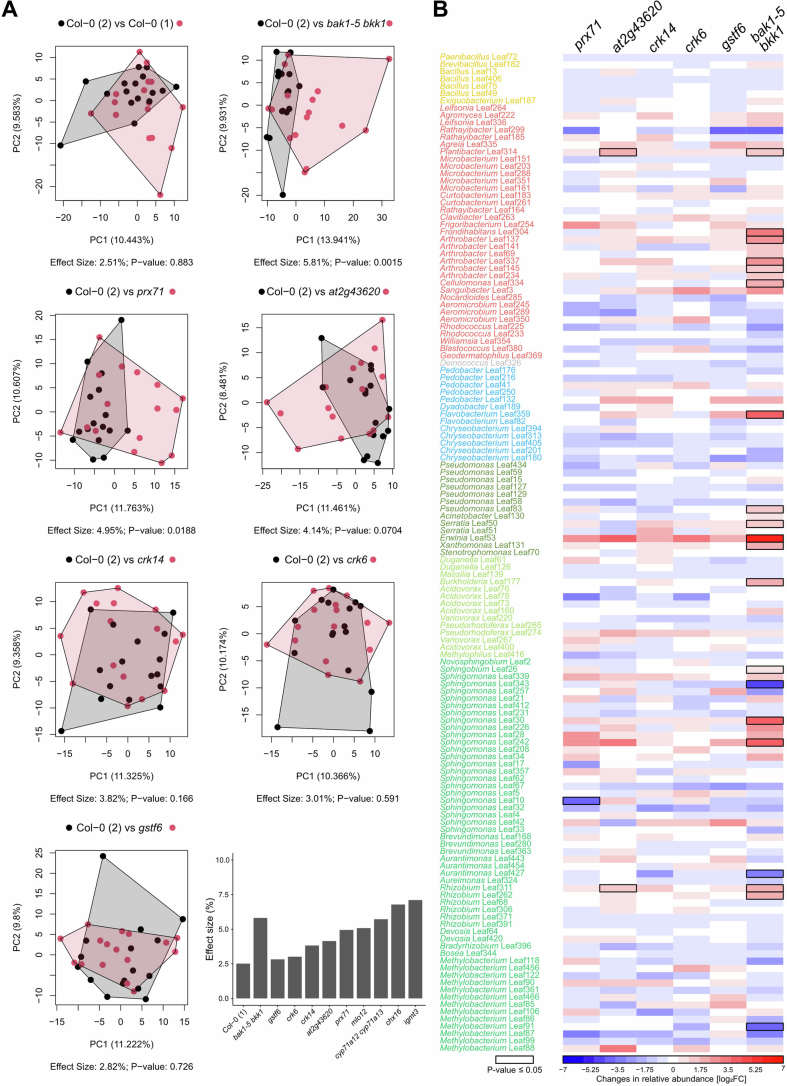
Microbiota composition analysis. (**A**) PCA of synthetic microbiota (SynCom-137) in *A. thaliana* Col-0 wild-type (independent, negative control), *bak1-5 bkk1* (positive control), and five GNSR mutant plant lines (indicated above plots) without significant changes compared to Col-0 wild-type. PC1 and PC2 are principal components 1 and 2 with their explained variance (%). Effect size and P-value were calculated by PERMANOVA (10^4^ permutations) and represent variance explained by genotype and corresponding statistical significance, respectively (Benjamini–Hochberg adjusted). Bar plot on the bottom right summarizes calculated effect sizes across genotypes, including those shown in Fig. 1. (**B**) Heatmap of log_2_FCs of abundance of microbiota strains in mutant plant lines shown in (**A**) relative to *A. thaliana* Col-0 wild-type. Rectangles outlined in black indicate statistically significant changes (P-value ≤ 0.05, two-sided Wald test, Benjamini–Hochberg adjusted). Strains are ordered according to phylogeny. (**A**, **B**) Data from one experiment with n = 16 plants per condition. Source data

Examination of the relative composition of strains revealed substantial shifts in leaf microbiota assembly on *igmt3*, *chx16*, *cyp71a12* *cyp71a13* and *mlo12* plants (effect sizes between 5.1% and 7.1%; *P* ≤ 0.01) (Fig. 1a and Extended Data Fig. 2). The overall change was similar to that of a *bak1-5* *bkk1* mutant (effect size of 5.8%, *P* ≤ 0.01; Extended Data Fig. 2) that is impaired in PTI signalling mediated by leucine-rich repeat PRRs^38^ and was included as a positive control^9^.

**Extended Data Fig. 3:**
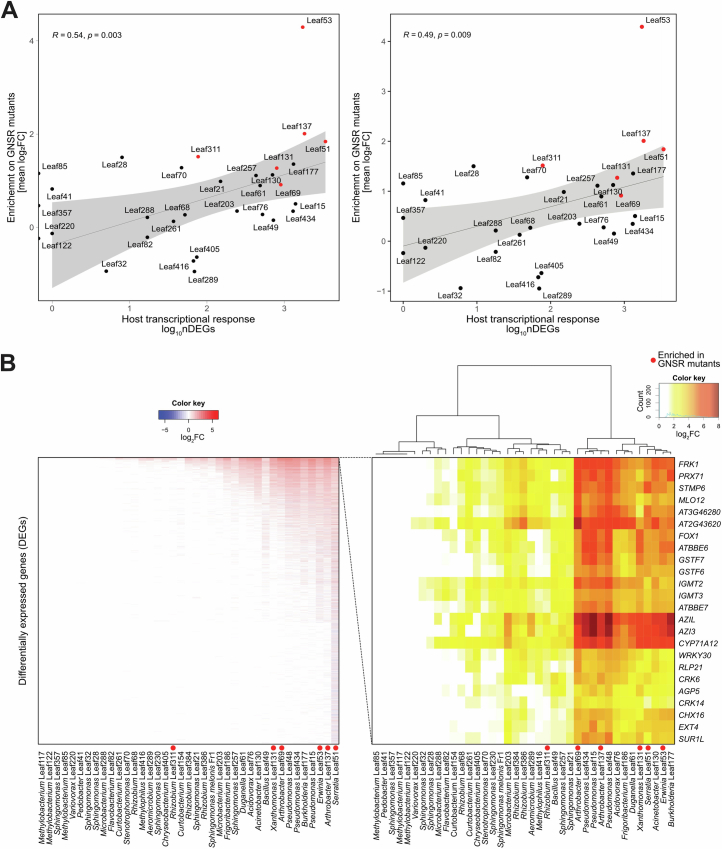
Relationship between effect of GNSR mutations on microbiota strains and elicited host response intensity. (**A**) Correlation analysis between mean log_2_FC in abundance of each strain across microbiota of GNSR mutants (relative to wild-type plants) and intensity of transcriptional plant response (in number of differentially expressed plant genes [nDEGs]), as determined by Maier et al. (2021, ref. ^21^), indicating that strains that benefit from lack of GNSR components tend to induce a strong host response. Regression shown in the left panel includes all strains, whereas in the right panel extreme cases Leaf53, Leaf85, Leaf357, and Leaf122 were excluded. *R* indicates Spearman’s correlation coefficient, *p* inidcates P-value. Shaded area depicts 95 % confidence interval. (**B**) As reference, plant response data were replotted from Maier et al. (2021, ref. ^21^) where extent of transcriptional reprogramming (left) and GNSR induction (right) in *A. thaliana* Col-0 wild-type was determined upon colonization by 39 diverse strains of the *At*-LSPHERE (x-axis). Heatmaps show mean log_2_FC of gene expression relative to axenic control plants. (**A**, **B**) Highlighted with a red dot are strains that were found to be consistently enriched in mutant plants lacking GNSR components, as shown in Fig. 1. Source data

When we analysed microbiota changes at the level of strain identity, 8–16% of strains had significantly altered relative abundances on *igmt3*, *chx16*, *cyp71a12* *cyp71a13* and *mlo12* mutant plants (*P* ≤ 0.05) (Fig. 1b). Of these, most strains (79%) were enriched relative to wild-type plants, and fewer were depleted (21%). Certain microbiota strains were consistently more abundant across the four GNSR mutants. These strains were *Leifsonia* Leaf336, *Curtobacterium* Leaf183, *Arthrobacter* Leaf137, *Arthrobacter* Leaf145, *Serratia* Leaf50 and *Rhizobium* Leaf311 (Fig. 1b). Moreover, bacteria closely related to these showed a tendency towards enrichment in GNSR mutants, notably members of the genus *Arthrobacter* (Leaf141, Leaf69 and Leaf337) and Gammaproteobacteria of the orders Enterobacteriales (*Serratia* Leaf51 and *Erwinia* Leaf53) and Xanthomonadales (*Xanthomonas* Leaf131). Of these strains, six had been analysed previously for transcriptional plant responses. Notably, except for *Rhizobium* Leaf311, the five other strains were among the strongest elicitors of the GNSR and overall plant response^21^ (Extended Data Fig. 3), potentially suggesting a negative feedback on bacterial colonization of leaves upon the induction of a plant response.

**Fig. 2:**
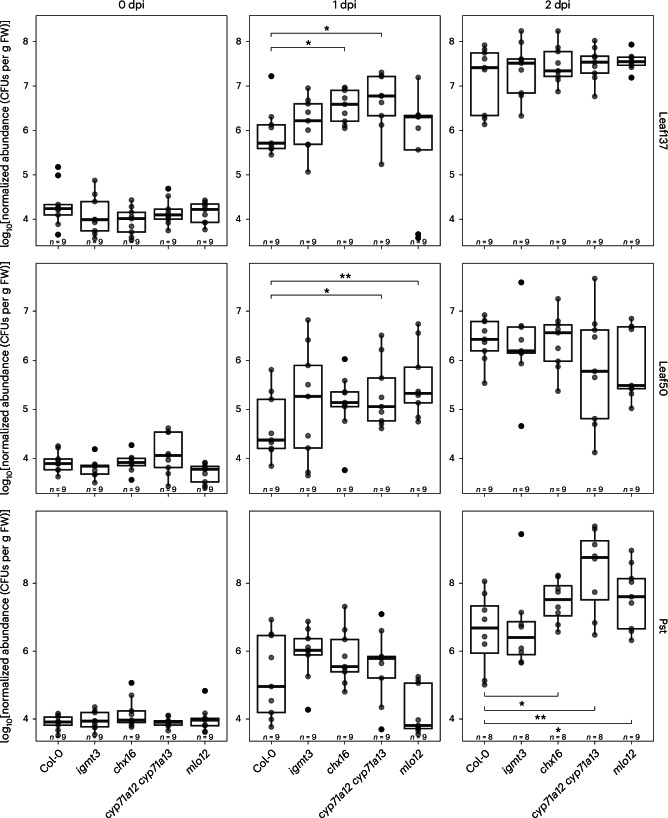
Colonization of GNSR mutant plants by leaf bacteria. Abundances of *Arthrobacter* Leaf137, *Serratia* Leaf50 and the foliar pathogen Pst on leaves, showing increased bacterial proliferation on plants with deficiencies in GNSR components. Colonization was monitored over the course of two days after inoculation of *A. thaliana* Col-0 wild-type plants and the indicated GNSR mutants (*x* axes) with bacterial suspensions at an OD_600_ of 0.00002. In each box plot, the horizontal bar indicates the median, the box edges indicate the first and third quartiles, and the whiskers indicate the smallest and largest values within 1.5× the interquartile range (IQR). The data are from one experiment with *n* = 8–9 plants per condition (indicated above each *x* axis). Statistical significance was assessed using one-sided *t*-tests relative to Col-0 (**P ≤ 0.01; *P ≤ 0.05). CFUs, colony-forming units; dpi, days post inoculation; FW, fresh weight. Source data

**Extended Data Fig. 4:**
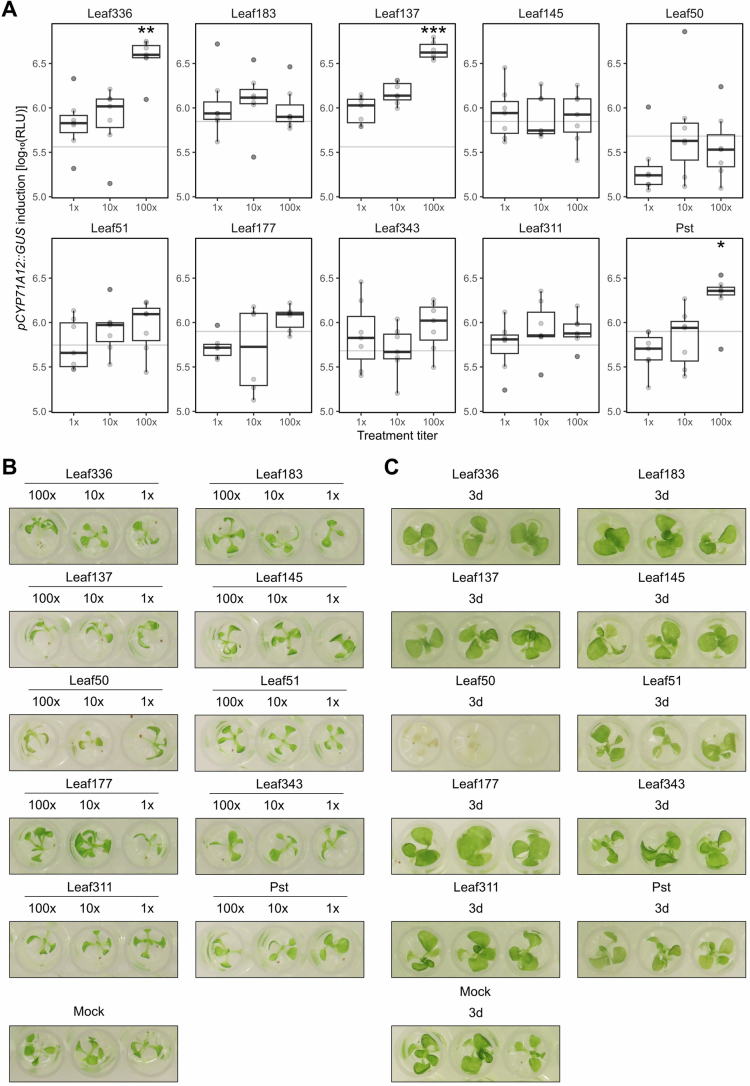
Screening microbiota strains with altered abundances in the microbiota of GNSR mutants for *CYP71A12* induction potential and plant phenotypes. (**A**) *CYP71A12* induction by nine microbiota strains and the foliar pathogen *P. syringae* pv. *tomato* DC3000 (Pst) upon exposure of *A. thaliana pCYP71A12::GUS* reporter seedlings to bacterial suspensions in a fluorometric assay screen for one day. Included were microbiota strains that were affected by mutations in GNSR components in a microbiota context (highlighted by grey diamonds in Fig. 1b). Vertical plot line indicates mean fluorescence of mock-treated plants on the same plate as the indicated treatment. Boxplot bars indicate median, hinges indicate first and third quartile, whiskers indicate smallest/largest value within 1.5 × IQR (interquartile range). Data from one experiment with n = 6–7 plants per condition (indicated above x-axis). Statistical significance was assessed by two-sided t-test with Bonferroni correction using titer “1×” as reference group (***: P-value ≤ 0.001, **: P-value ≤ 0.01, and *: P-value ≤ 0.05). (**B**) Representative images of *pCYP71A12::GUS* reporter plants one day after treatment with indicated bacteria. Pictures were taken immediately before performing the fluorometric assay. (**C**) Representative images of *pCYP71A12::GUS* reporter plants three days after treatment (3 d), where disease phenotype caused by *Serratia* Leaf50 is enhanced. Treatment suspensions of leaf microbiota strains or Pst were prepared at a 10× titer. (**A**, **B**) Indicated titers of 1×, 10×, and 100× correspond to bacterial treatment suspensions adjusted to an OD_600_ of 0.002, 0.02, and 0.2 before treatment, respectively. Source data

We thus speculated that the changes in community structure in GNSR mutants (Fig. 1) might be the result of impaired colonization by strains that trigger a strong plant response, which tended to be enriched in GNSR mutant microbiota (Extended Data Fig. 3). To address this hypothesis, we assessed whether the population sizes and thus colonization dynamics of microbiota strains in the four GNSR mutant lines *igmt3*, *chx16*, *cyp71a12* *cyp71a13* and *mlo12* were increased at early stages of colonization compared with wild-type plants. We chose to monitor leaf colonization by the commensal *Arthrobacter* Leaf137, the opportunistic pathogen *Serratia* Leaf50 (ref. ^4^) and the foliar pathogen Pst after validating that the exposure of seedlings to these strains caused substantial induction of *CYP71A12* or disease^4^ (Extended Data Fig. 4). We observed a significant increase in the abundance of all three strains on GNSR mutants. Within the first day, *Arthrobacter* Leaf137 colonized *chx16* and *cyp71a12* *cyp71a13* mutants to approximately 7-fold and 11-fold higher levels than wild-type plants, respectively. Similarly, *Serratia* Leaf50 reached approximately 5-fold and 9-fold higher abundances on *cyp71a12* *cyp71a13* and *mlo12* mutants than on wild-type plants, respectively (Fig. 2). These effects on bacterial population sizes were transient, with comparable bacterial abundances on GNSR mutants and wild-type plants two days after inoculation. Pst exhibited increased abundances ranging from 7-fold in *chx16* and *mlo12* to up to 120-fold in *cyp71a12* *cyp71a13* after two days of colonization, relative to wild-type plants (Fig. 2). While these GNSR components were previously implicated in defence against pathogens, particularly *CYP71A12* (refs. ^21,22,31,32^), it was remarkable that they affected diverse members of the leaf microbiota. Such colonization advantages conferred to individual strains by the lack of GNSR components could result in increased relative abundances during early colonization that translate into the altered microbiota compositions observed in GNSR mutant plants (Fig. 1). Together, these observations support the notion that GNSR genes, which are induced by the microbiota, feed back to inhibit colonization by individual strains with diverse lifestyles and contribute to microbiota homeostasis.

### GNSR induction increases during bacterial colonization

Both GNSR intensity and overall plant response are positively correlated with bacterial population size^21^. Together with the enrichment of strains that elicit a strong host response during microbiota assembly in GNSR mutants (Fig. 1 and Extended Data Fig. 3) and the feedback onto bacterial leaf colonization (Fig. 2), this suggests a relationship between the extent of exposure to individual strains and GNSR induction in the plant.

To investigate the dynamics of GNSR induction, we inoculated axenic *A. thaliana* seedlings with two leaf microbiota strains of different phyla and assessed bacterial colonization levels and induction of GNSR genes over the course of nine days. We chose *Arthrobacter* Leaf137 and *Rhizobium* Leaf68, which cause strong and moderate reprogramming of the plant transcriptome, respectively^21^. *Arthrobacter* Leaf137 was of particular interest due to its increased abundance on GNSR mutants in a microbiota context (Fig. 1b) and in early mono-association (Fig. 2). We monitored the GNSR using real-time quantitative PCR (RT-qPCR) to measure the expression of *CYP71A12* (*AT2G30750*), as this gene is the best representative of the overall host response, apart from contributing to microbiota assembly and affecting colonization by individual strains^21^ (Figs. 1 and 2). As a second GNSR gene, we selected *AZIL* (*AT4G12500*), whose encoded protein belongs to the AZI family of lipid transfer proteins. This protein family contributes to systemic resistance against pathogens upon azelaic acid perception^24,39^.

**Fig. 3:**
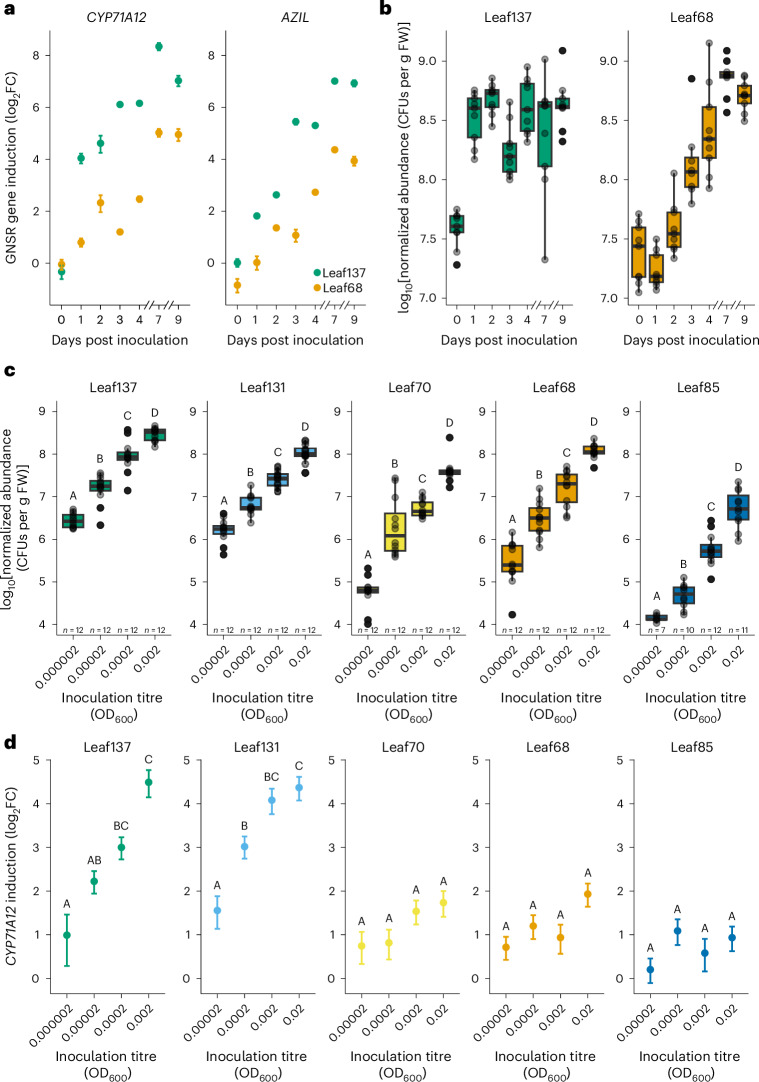
GNSR induction during bacterial colonization of leaves. **a**, Induction of the GNSR genes *CYP71A12* (left) and *AZIL* (right) (*y* axes) in *A. thaliana* Col-0 wild-type plants in response to *Arthrobacter* Leaf137 and *Rhizobium* Leaf68 in mono-association over the course of nine days (*x* axes), showing a gradual increase of expression. Inoculation was at 0 d at an OD_600_ of 0.02. The points indicate mean log_2_FCs of normalized gene expression relative to axenic control plants. The error bars indicate propagated standard error. The data are from one experiment with *n* = 1 replicates comprising 18 pooled plants per condition, measured in duplicate. **b**, Normalized abundance of *Arthrobacter* Leaf137 and *Rhizobium* Leaf68 (*y* axes) in planta during nine days of colonization (as in **a**). In each box plot, the horizontal bar indicates the median, the box edges indicate the first and third quartiles, and the whiskers indicate the smallest and largest values within 1.5× the IQR. The data are from one experiment with *n* = 9 plants per condition. The experiments in **a**,**b** were reproduced in an independent biological replicate, and similar values were obtained (Extended Data Fig. 5). **c**, Normalized abundance (*y* axes) of five microbiota strains (indicated above each plot) after one day of colonization as a function of inoculum density (*x* axes), showing titrated bacterial colonization levels. In each box plot, the horizontal bar indicates the median, the box edges indicate the first and third quartiles, and the whiskers indicate the smallest and largest values within 1.5× the IQR. The data are from one experiment with *n* = 7–12 plants per condition (indicated above the *x* axes). **d**, Induction of the GNSR gene *CYP71A12* (*y* axes) after one day of colonization by five microbiota strains inoculated at different densities (*x* axes) (as in **c**), showing dose-responsive induction according to bacterial population size. The points indicate mean log_2_FCs of normalized gene expression relative to axenic control plants. The error bars indicate propagated standard error. The data are from one experiment with *n* = 4 replicates comprising a total of 16–20 plants per condition, measured in duplicate. In **c**,**d**, the letters above the points indicate significance levels obtained from one-way ANOVA with Tukey’s post-hoc test. Source data

**Extended Data Fig. 5:**
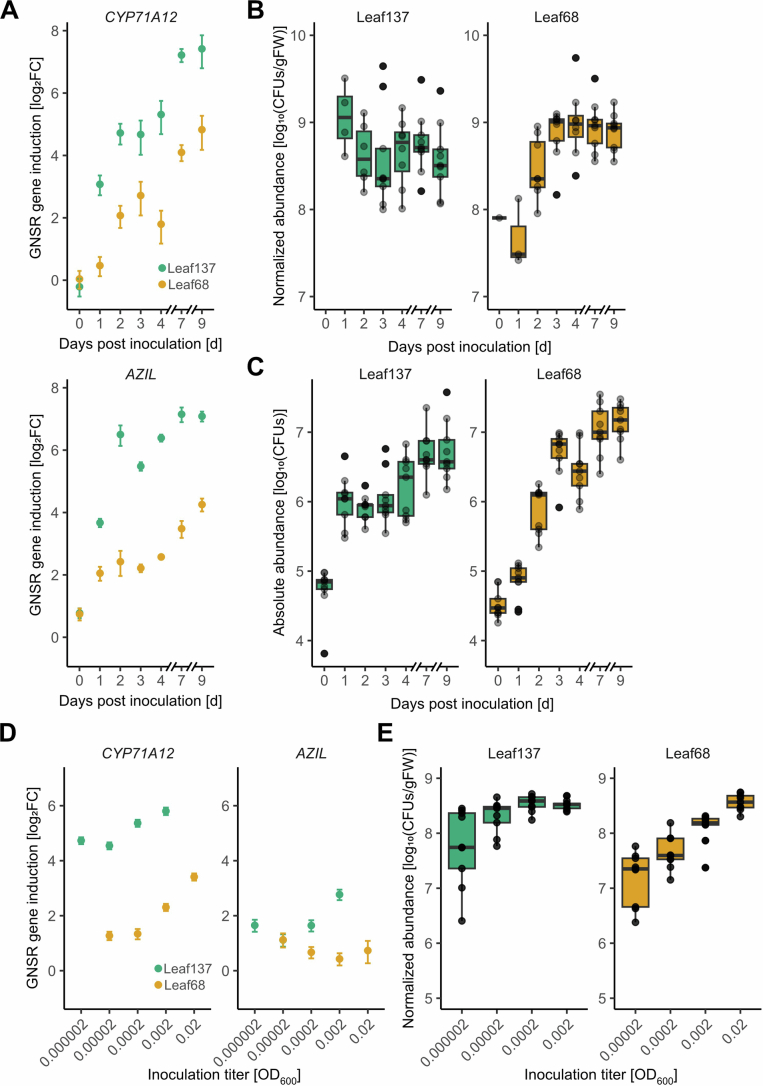
GNSR induction during bacterial colonization. (**A**–**C**) Validation of data shown in Fig. 3. (**A**) Induction dynamics of GNSR genes *CYP71A12* (top) and *AZIL* (bottom) in *A. thaliana* Col-0 wild-type plants during colonization by *Arthrobacter* Leaf137 and *Rhizobium* Leaf68 in mono-association over the course of nine days. Inoculation at 0 d with inoculum density of OD_600_ = 0.02. Points indicate mean log_2_FCs of normalized gene expression relative to axenic control plants. Error bars indicate propagated standard error. Data from one experiment with n = 1 replicates comprising 18 pooled plants per condition measured in duplicate. (**B**) Normalized and (**C**) absolute abundance of *Arthrobacter* Leaf137 and *Rhizobium* Leaf68 *in planta* during nine days of colonization. Bars indicate median, hinges indicate first and third quartile, whiskers indicate smallest/largest value within 1.5 × IQR (interquartile range). Data from one experiment with n = 9 plants per condition. (**D**) Induction of GNSR genes *CYP71A12* and *AZIL* (y-axis) by *Arthrobacter* Leaf137 and *Rhizobium* Leaf68 two days after inoculation at indicated density (x-axis). Points indicate mean log_2_FCs of normalized gene expression relative to axenic control plants. Error bars indicate propagated standard error. Data from one experiment with n = 1 replicates comprising 12 pooled plants per condition measured in duplicate. (**E**) Normalized bacterial abundance (y-axis) *in planta* two days after inoculation at indicated inoculum density (x-axis). Leaf microbiota strains (as in **D**) indicated above each plot. Bars indicate median, hinges indicate first and third quartile, whiskers indicate smallest/largest value within 1.5 × IQR. Data from one experiment with n = 9 plants per condition. Source data

We found that *Rhizobium* Leaf68 increased in abundance during the first four to seven days of colonization until reaching the carrying capacity of the plant. *Arthrobacter* Leaf137 reached its maximal population size only one day after inoculation, indicating that the strain colonizes leaves remarkably fast (Fig. 3 and Supplementary Fig. 1), consistent with the data shown in Fig. 2. The difference in colonization dynamics between the two strains was reflected in the induction of *CYP71A12* and *AZIL* (Fig. 3a). *Arthrobacter* Leaf137 caused substantial induction of *CYP71A12* and *AZIL* as early as one day after inoculation, which continued to rise to a 128-fold increase in expression relative to axenic controls. Expression of the GNSR genes was slower in response to *Rhizobium* Leaf68 colonization and reached lower final levels, which were 16-fold (*AZIL*) and 32-fold (*CYP71A12*) greater than those of axenic controls. Importantly, both bacterial abundance and GNSR induction levels after nine days of colonization were consistent with previous work^21^ and comparable between independent experiments (Fig. 3a,b, Extended Data Fig. 5a–c and Supplementary Fig. 1). Our data thus reveal that GNSR induction increases with bacterial abundance and time, suggesting that plant response intensity during colonization is driven by the degree and duration of exposure to bacteria.

To investigate possible dose effects, we inoculated wild-type *A. thaliana* seedlings with five microbiota strains at various titres, spanning four orders of magnitude, and assessed bacterial abundance and *CYP71A12* induction levels after one day of colonization. We reasoned that low initial bacterial populations would not propagate sufficiently to reach carrying capacity within this period, on the basis of data where longer colonization probably obscured dose effects (Extended Data Fig. 5d,e). In addition to *Arthrobacter* Leaf137 and *Rhizobium* Leaf68, we included *Xanthomonas* Leaf131, *Stenotrophomonas* Leaf70 and *Methylobacterium* Leaf85, representing microbiota members that elicit host transcriptional responses to varying degrees upon extended colonization^21^ (Extended Data Fig. 3).

Bacterial colonization levels were indeed distinct depending on inoculation titre, with differences between the lowest and highest population sizes ranging up to approximately 300-fold (Fig. 3c). The strong response elicitors *Arthrobacter* Leaf137 and *Xanthomonas* Leaf131 caused increasing *CYP71A12* induction with rising inoculation titre, ranging from around 2-fold to 16-fold greater expression than in axenic controls (Fig. 3d), reflecting corresponding levels of colonization. *Stenotrophomonas* Leaf70 and *Rhizobium* Leaf68 exhibited a tendency of increasing *CYP71A12* induction with population size, with approximately 2-fold and 4-fold induction at the lowest and highest titres, respectively (Fig. 3d). In contrast, *Methylobacterium* Leaf85 failed to induce *CYP71A12* above low levels (that is, log2 transformed fold change (log_2_FC) > 1 compared with axenic plants) (Fig. 3d), in line with previous data^21^. These findings suggest that the host response during early colonization is dynamically responsive to bacterial abundance.

**Fig. 4:**
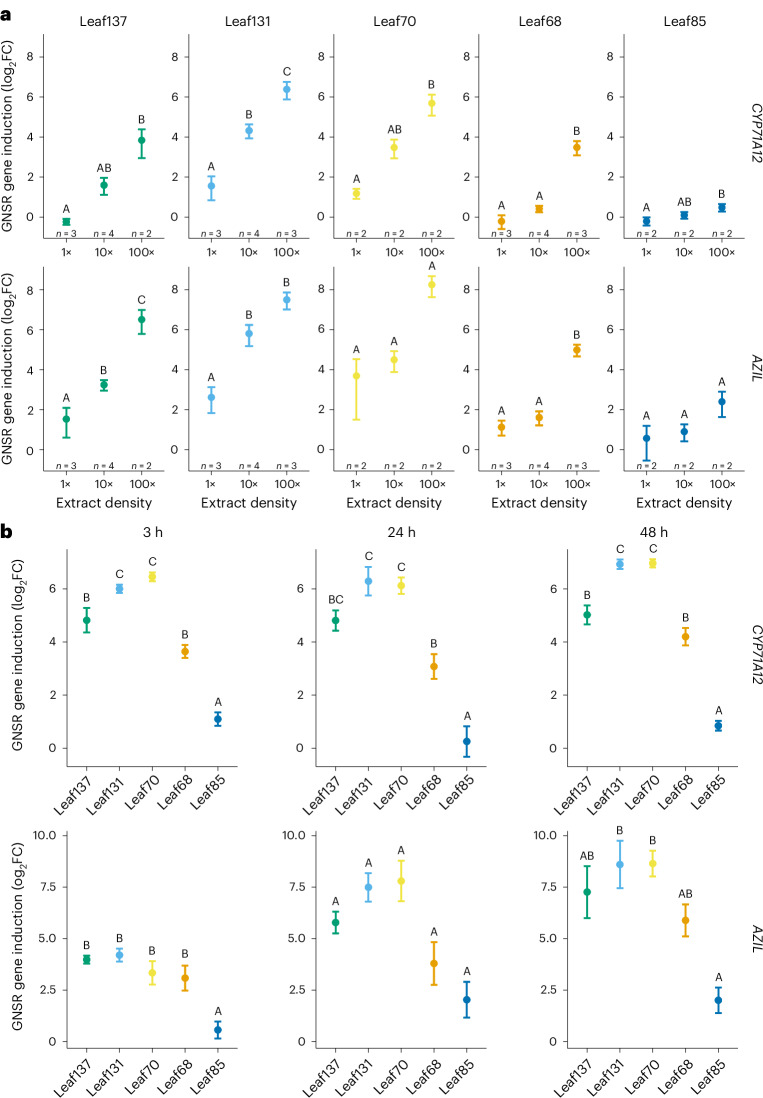
Responsiveness of GNSR genes to boiled culture extracts of leaf microbiota strains. **a**, Induction levels of the GNSR genes *CYP71A12* (top) and *AZIL* (bottom) by bacterial culture extracts of five leaf microbiota strains two days after treatment exhibit dose dependency. Bacterial suspensions at the indicated densities (*x* axes, where 1×, 10× and 100× correspond to OD_600_ of 0.02, 0.2 and 2, respectively, before boiling) were boiled and applied onto *A. thaliana* seedlings. The data are from *n* = 2–4 independent experiments (indicated above the *x* axis), each comprising 18 pooled plants per condition measured in duplicate. **b**, Induction of *CYP71A12* (top) and *AZIL* (bottom) by bacterial culture extracts at three early time points (3, 24 and 48 h post treatment, indicated above the plots), showing varying dynamics of gene expression between extracts and GNSR genes. Bacterial suspensions of five leaf microbiota strains at an OD_600_ of 2 (corresponding to an extract density of 100× in **a**) were boiled and applied onto *A. thaliana* seedlings. The points indicate mean log_2_FCs of normalized gene expression relative to axenic control plants. The error bars indicate propagated standard error. The data are from *n* = 3 independent experiments, each comprising 18 pooled plants per condition measured in duplicate. In **a**,**b**, the letters above the points indicate significance levels obtained from one-way ANOVA with Tukey’s post-hoc test. Source data

In addition, we analysed GNSR induction strength and dynamics within two days of treatment of seedlings with bacterial extracts, a commonly used approach to monitor host response dynamics without confounding effects of bacterial growth^40–42^. Boiled culture extracts of *Arthrobacter* Leaf137, *Xanthomonas* Leaf131, *Stenotrophomonas* Leaf70 and *Rhizobium* Leaf68 induced *CYP71A12* and *AZIL* in a dose-dependent manner two days after treatment (Fig. 4a), as expected from their induction potentials during early colonization (Fig. 3 and Extended Data Fig. 5). Extracts derived from *Methylobacterium* Leaf85 showed a weak response, consistent with data from live bacteria^21^ (Fig. 3c,d). Interestingly, *Stenotrophomonas* Leaf70 extracts induced *CYP71A12* and *AZIL* strongly, in contrast to the weak plant response observed upon its colonization^21^ (Figs. 3c,d and 4a).

To analyse induction dynamics, we fixed bacterial extract concentrations (based on adjusted optical density at 600 nm (OD_600_)) and compared GNSR induction between strains at three time points. Most extracts caused substantial *CYP71A12* induction within 3 h (log_2_FC ≥ 4, except *Methylobacterium* Leaf85) (Fig. 4b). Extracts of *Xanthomonas* Leaf131 and *Stenotrophomonas* Leaf70 induced *CYP71A12* most strongly, followed by *Arthrobacter* Leaf137 and *Rhizobium* Leaf68, and lastly *Methylobacterium* Leaf85 extracts (log_2_FC < 2). Induction levels remained largely constant up to 48 h after treatment (∆log_2_FC ≤ 1 between time points within strains) (Fig. 4b). In contrast, *AZIL* induction exhibited little differences between extracts within 3 h apart from *Methylobacterium* Leaf85. Differences in induction were apparent 24 h after extract treatments and became discernible after 48 h (Fig. 4b). The final levels of *CYP71A12* and *AZIL* induction were in line with previous observations at the corresponding time point (Fig. 4a) and matched the expected induction potentials observed for responses to live bacteria from transcriptome sequencing^21^ (Fig. 3 and Extended Data Fig. 3). An exception was *Stenotrophomonas* Leaf70, whose extracts exhibited not only similar levels of GNSR induction as those of *Xanthomonas* Leaf131 (Fig. 4a) but also similar dynamics (Fig. 4b). Additionally, *AZIL* induction levels at 48 h were not reached before the endpoint of measurements and might therefore not reflect final magnitudes. This suggests that *AZIL* expression upon non-self perception is delayed compared with that of *CYP71A12*, probably explaining its poor dose response during early colonization in previous experiments (Extended Data Fig. 5d,e). Taken together, our findings show that early strength and dynamics of GNSR induction are strain-specific and differ between GNSR genes, highlighting a dynamic response to microbiota signals.

### The GNSR overlaps with PTI responses

**Extended Data Fig. 6:**
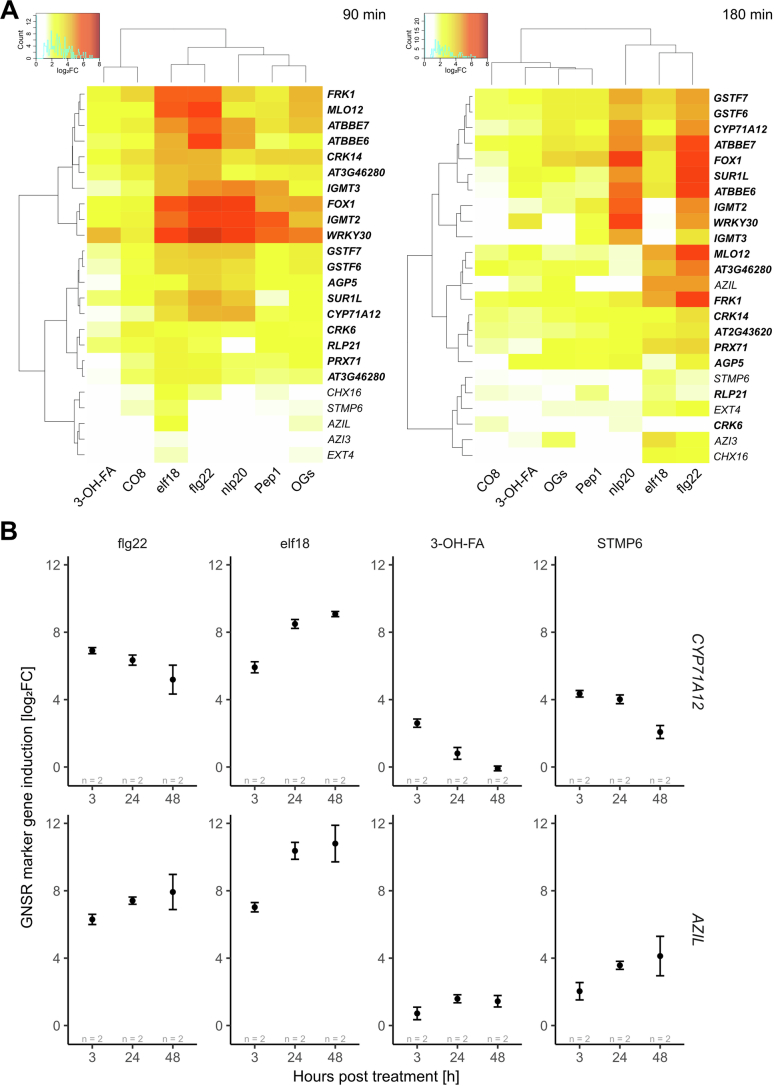
Examination of PTI elicitors and GNSR induction. (**A**) Induction of GNSR genes (y-axis) at 90 min (left) and 180 min (right) after treatment with diverse MAMPs, PAMPs, or DAMPs (x-axis), derived from bacteria, fungi, insects, or plants, as reported by Bjornson et al. (2021, ref. ^29^). Color indicates log_2_FC as shown in the key. GNSR genes that were found to be commonly induced by all elicitors within 3 h after treatment are highlighted in bold. Conditions clustered by Ward’s method. (**B**) Validation of *CYP71A12* (top) and *AZIL* (bottom) induction (y-axis) by select MAMPs and DAMPs at three early timepoints. Seedlings were inoculated with 15 µl of elicitors diluted to 10 µM (flg22, elf18, 3-OH-FA) or 100 µM (STMP6). Points indicate mean log_2_FCs of normalized gene expression relative to axenic control plants, error bars indicate propagated standard error. Data from n = 2 independent experiments, each comprising 18 pooled plants per condition measured in duplicate. Source data

Microbial extracts have been known to harbour immunogenic bacterial elicitors since the initial discovery of flg22 (refs. ^41,43^). PTI responses to individual MAMPs or DAMPs were recently systematically characterized at the transcriptional level in *A. thaliana*^29^. This study revealed a large overlap in shared gene expression changes upon the perception of seven known elicitors derived from bacteria, fungi, oomycetes and plants, of approximately 1,000 genes within three hours of application^29^. A major part of the PTI response is congruent to that induced rapidly by other stresses and thus corresponds to a general stress response (GSR)^29,44^. Yet, genes of the GSR and the microbiota-induced GNSR^21^ have not yet been linked. We therefore re-analysed the GSR and found that it comprised 19 of 24 GNSR genes (Extended Data Fig. 6a), indicating a centralized response to non-self perception. The remaining GNSR genes were expressed only upon the perception of individual elicitors. Interestingly, these 19 GNSR genes were among the first genes to be induced upon MAMP/DAMP perception, but they also remained expressed steadily thereafter^29^. In contrast to the GSR, a small set of core immunity response (CIR) genes is specifically induced by elicitors and not by other stresses^29^. However, the CIR and the GNSR share only one gene in common (*RLP21*), probably indicating that elicitor-specific responses constitute early events in PTI, followed by gene expression changes that integrate them with more complex microbiota signals at later stages.

Because PTI-associated transcriptional reprogramming was determined upon treatment of *A. thaliana* seedlings grown in liquid medium^29^, we validated the induction of GNSR by different immunogenic patterns in the agar-based gnotobiotic plant growth system used here by applying elicitors onto leaves, analogously to inoculation with bacteria. As treatments, we included the bacterial elicitors flg22, elf18 and 3-OH-FA, and plant-derived STMP6 (also called SCOOP39), a peptide encoded by a GNSR gene that has been implicated in plant defence and is probably perceived as a perturbed-self signal^45,46^. We measured the induction of the GNSR genes *CYP71A12* and *AZIL* after elicitor treatment at previously analysed time points—that is, 3 h (ref. ^29^) and 24 and 48 h (Fig. 3 and Extended Data Fig. 5). Both flg22 and elf18 induced the GNSR genes strongly, while plants treated with STMP6 and 3-OH-FA exhibited a lower degree of induction (Extended Data Fig. 6b). In summary, we found a convergence of signals in PTI-mediated transcriptional reprogramming encompassing the GNSR. This reinforces the notion of a convergent non-self immune response.

**Fig. 5:**
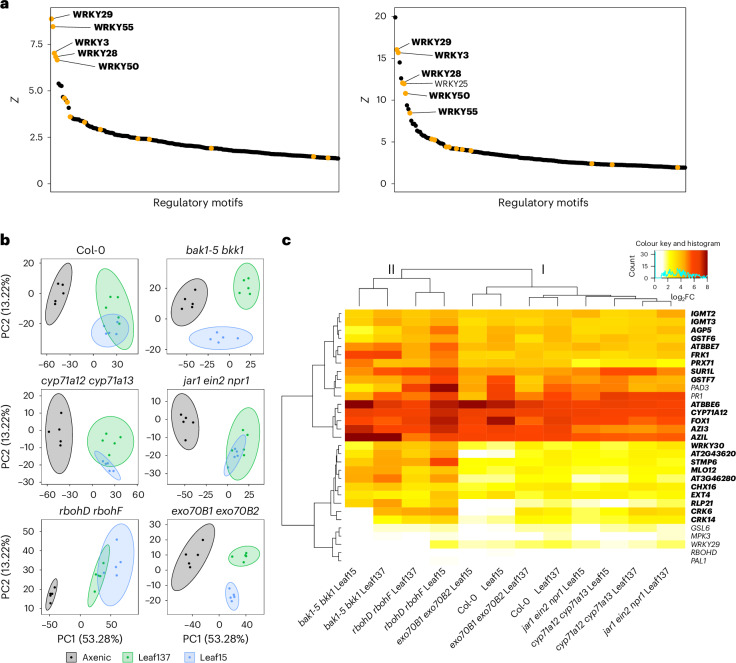
Analysis of signalling networks and transcriptional host responses to leaf microbiota strains. **a**, Regulatory networks in *A. thaliana* were inferred with ISMARA^47^ using RNA sequencing data of responses to leaf microbiota strains (reported in Maier et al.^21^) (left) and to diverse MAMPs/DAMPs (reported in Bjornson et al.^29^) (right), indicating that transcriptional reprogramming upon bacterial colonization and elicitor perception is predicted to be driven by overlapping WRKY TFs. The regulatory motifs (*x* axes, cut-off at 200) are ordered by significance (*Z* value, *y* axes). Regulatory motifs targeted by WRKY TFs are highlighted in yellow, and the top five motifs of the left panel are shown in bold. **b**, PCA of differentially expressed genes (*P* ≤ 0.01, two-sided Wald test with Benjamini–Hochberg correction, filtered for |log_2_FC| ≥ 1 relative to axenic control in at least one condition) in response to *Arthrobacter* Leaf137 and *Pseudomonas* Leaf15 within genotypes (indicated at the top of each plot). *Arthrobacter* Leaf137 and *Pseudomonas* Leaf15 cause distinct transcriptional responses depending on the genetic background of the host. **c**, Heat map of log_2_FCs of GNSR genes (bold) and selected PTI marker genes (non-bold) (vertical axis) in Col-0 wild-type plants or mutants colonized by the indicated strains (horizontal axis) relative to the corresponding genotype under axenic conditions. *Arthrobacter* Leaf137 and *Pseudomonas* Leaf15 consistently induced the GNSR in plants deficient in key immunity signalling components. Genes were filtered for *P* ≤ 0.01 (two-sided Wald test with Benjamini–Hochberg correction). Colour indicates the extent of fold change as indicated by the colour key. Conditions and genes are clustered by Ward’s method, as indicated by the trees. Main condition clusters are designated as I and II. In **b**,**c**, the data are from *n* = 5 independent experiments, each comprising 18 pooled plants per condition, sampled at nine days post inoculation. Source data

We speculated that this response would be orchestrated by a common regulatory network. To address this hypothesis, we inferred the regulatory networks from the available RNA sequencing data^21^ using the Integrated System for Motif Activity Response Analysis (ISMARA)^47^, covering 573 regulatory motifs (Supplementary Table 2). The five most significantly detected motifs were all binding sites of WRKY family transcription factors (TFs), which are broadly implicated in mediating abiotic and biotic stresses in plants^48,49^. These were WRKY29, WRKY55, WRKY3, WRKY28 and WRKY50 (*Z* = 8.9, 8.4, 7.0, 6.8 and 6.7, respectively) (Fig. 5a). We found a significant correlation between measured expression levels of *WRKY29* (*ρ* = 0.82), *WRKY55* (*ρ* = 0.88) and *WRKY28* (*ρ* = 0.51) and the inferred activity of their corresponding regulatory motifs (Supplementary Fig. 2a,b,d), suggesting that these TFs are positive regulators of gene expression. This correlation was weaker for *WRKY50* and *WRKY3* (*ρ* = 0.36 and 0.17, respectively) (Supplementary Fig. 2c,e). Interestingly, 21 of 24 GNSR genes occurred among the 3,986 predicted target genes of these WRKY TFs, representing a significant enrichment relative to other genes as potential targets (Fisher’s exact test, *P* < 0.001) (Supplementary Table 3). All five WRKY TFs were predicted to target the GNSR gene *WRKY30*, highlighting its possible role as a transcriptional regulator of the GNSR, as suggested previously^21^. Moreover, analysis of the RNA data obtained from transcriptional responses to MAMPs and DAMPs^29^ also indicated significant roles of the same five WRKY TFs (Fig. 5a and Supplementary Table 4), as their binding sites occurred among the 11 most important predicted regulatory motifs.

We observed that leaf microbiota strains and their boiled culture extracts induce GNSR genes in a dose-dependent and dynamic manner (Figs. 3 and 4 and Extended Data Fig. 5). Moreover, this immune response to leaf bacteria, their culture extracts and individual MAMPs converges^29^ (Fig. 5a and Extended Data Fig. 6), provoking the question whether GNSR gene induction depends on canonical immunity pathways that were previously extensively characterized in response to individual MAMP exposure^14,15^.

To identify the components required for response elicitation upstream of GNSR genes, we tested a set of *A. thaliana* mutants with deficiencies in defence-related signalling pathways. We monitored plant gene expression in response to colonization by *Arthrobacter* Leaf137 and *Pseudomonas* Leaf15, a representative of a different phylum that showed a consistent and particularly strong host response^21^. Plant responses were recorded by RNA sequencing of leaves sampled nine days after inoculation. In addition to mutants in BAK1, BKK1 and CYP71A12, we included JAR1, EIN2 and NPR1, which are involved in the regulation of defence-related hormone signalling and systemic acquired resistance (SAR); RBOHD and RBOHF, two NADPH oxidases that produce reactive oxygen species (ROS) in response to pathogens; EXO70B1 and EXO70B2, components linked to the exocytosis of defence-related proteins and receptors; and CYP71A13, which like CYP71A12 contributes to the synthesis of specialized indole-derived secondary metabolites foremost associated with plant defence^36,50–52^.

**Extended Data Fig. 7:**
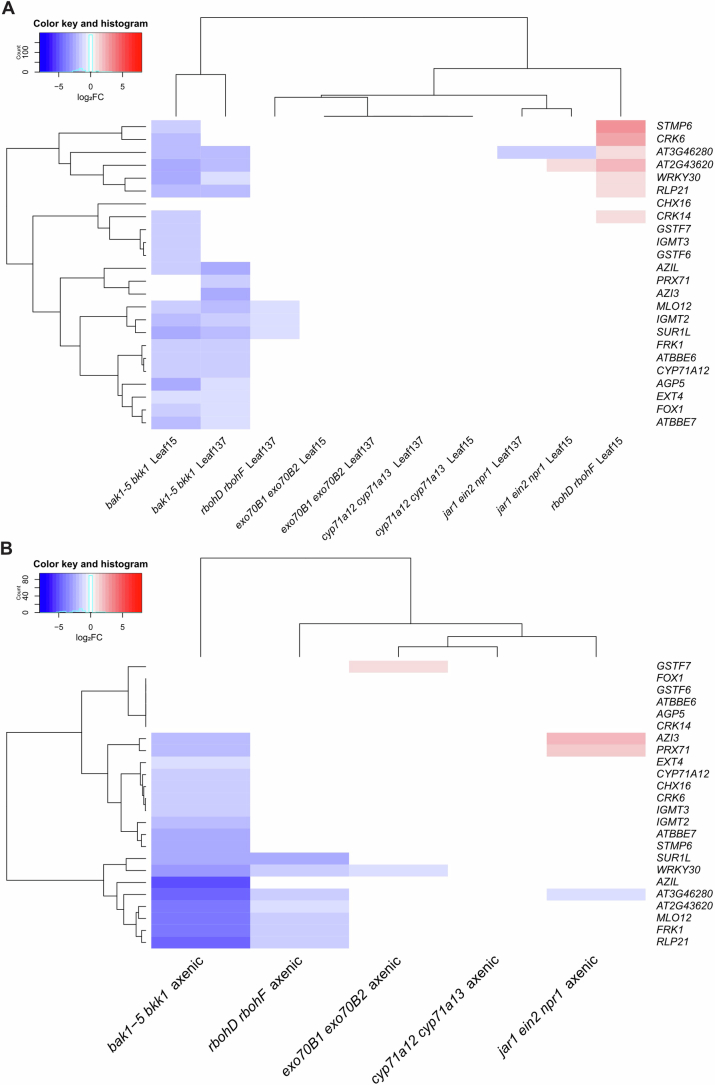
Comparison of GNSR expression in immunity and signalling mutants to inoculated and axenic *A. thaliana* Col-0 wild-type. (**A**) Heatmap of log_2_FCs of GNSR genes (y-axis) in mutant plant lines (x-axis) upon colonization by *Arthrobacter* Leaf137 or *Pseudomonas* Leaf15 relative to wild-type plants under the same treatment condition, showing altered expression levels in *bak1-5 bkk1* and *rbohD rbohF*. (**B**) Heat map of log_2_FCs of GNSR genes (y-axis) in axenic mutant plant lines (x-axis) relative to axenic wild-type plants, showing altered basal GNSR expression in *bak1-5 bkk1* and to a lesser extent in *rbohD rbohF* even in absence of bacteria. (**A**, **B**) Genes were filtered for P-value ≤ 0.01 (two-sided Wald test with Benjamini-Hochberg correction). Color indicates extent of induction as indicated by respective color key. Conditions clustered by Ward’s method. Data from n = 5 independent experiments, each comprising 18 pooled plants per condition, sampled at nine days post inoculation. Source data

Differentially expressed genes upon bacterial colonization were identified by comparison with mock-treated plants of the same genotype (adjusted *P* ≤ 0.01, |log_2_FC| ≥ 1 in at least one condition). Principal component analysis (PCA) of differentially expressed genes (based on gene expression counts normalized by variance-stabilized transformation^53^) across plant genotypes within bacterial treatments revealed that host responses to *Arthrobacter* Leaf137 and *Pseudomonas* Leaf15 in *bak1-5* *bkk1* and *rbohD* *rbohF* were distinct from those in wild-type plants (Supplementary Fig. 3). Interestingly, while *Arthrobacter* Leaf137 caused similar responses in the two mutant lines, *Pseudomonas* Leaf15 elicited different responses in *bak1-5* *bkk1* than in *rbohD* *rbohF*. It is unlikely that these observed disparities in transcriptional responses are caused by the minor differences in bacterial abundance between wild-type and mutant plant lines (Supplementary Fig. 4). Moreover, we found that *Arthrobacter* Leaf137 and *Pseudomonas* Leaf15 caused overlapping (Col-0, *cyp71A12* *cyp71A13*, *jar1* *ein2* *npr1* and *rbohD* *rbohF*) and distinct responses (*bak1-5* *bkk1* and *exo70B1* *exo70B2*) depending on plant genotype (Fig. 5b). Together, this suggests that the host response depends not only on the bacterial strains but also on the genetic background of the host. Regarding the expression of GNSR genes, however, the bacterial treatments elicited only slightly altered patterns of GNSR induction in some mutant plant lines compared with the wild type, resulting in two main clusters that differed in the overall strength of induction (Fig. 5c; the clusters are designated as I and II). Most mutant plant lines clustered with wild-type plants (cluster I, median log_2_FC = 3.5), including *cyp71A12* *cyp71A13*, *jar1* *ein2* *npr1* and *exo70B1* *exo70B2*. In contrast, a stronger GNSR was elicited in *bak1-5* *bkk1* and *rbohD* *rbohF*, distinct from all other conditions (cluster II, median log_2_FC = 4.4), which is congruent with the PCA (Supplementary Fig. 3). The observation that the GNSR was robustly induced in plants deficient in key immunity or signalling components (Fig. 5c and Supplementary Fig. 5a) suggests redundancy in the underlying signalling pathways. We extracted 11 additional genes from our dataset that are commonly used as PTI markers to contrast the observed expression patterns with the GNSR^50,54–59^. Most of these PTI genes exhibited significantly lower induction levels than the GNSR genes (*GSL6*, *MPK3*, *WRKY29*, *PAL1* and *RBOHD*) or were not differentially regulated in response to bacterial colonization (*GSL5*, *MPK6*, *PAL2* and *PHI-1*). Only two genes, *PR1* and *PAD3*, were consistently induced across conditions and clustered together with the GNSR genes (Fig. 5c and Supplementary Fig. 5b). This was interesting, as PAD3 is a cytochrome P450 monooxygenase like CYP71A12 and also contributes to the production of defence-related phytoalexins upon pathogen encounter^60,61^, highlighting the role of tryptophan-derived secondary metabolism in plant–microbiome interactions. To widen the scope of signalling pathways that could contribute to the GNSR, we probed GNSR induction by RT-qPCR in higher-order mutants with broader deficiencies in PTI signalling, upstream of the components tested above. These mutants were deficient in the perception of flagellin (*fls2*), elongation factor Tu (*efr*) and/or peptidoglycan (*cerk1*) elicitors, or compromised in immune signal activation downstream of various PRRs (*bak1-5* *bkk1* and/or *cerk1*)^62–66^. We also included the vesicle trafficking mutant *min7*, which was recently shown to contribute to microbiota homeostasis and pathogen resistance^8,65^. Strikingly, even in the absence of multiple signalling components central to PTI, the GNSR genes *CYP71A12* and *AZIL* were induced by *Arthrobacter* Leaf137 (Supplementary Fig. 6). This lends support to the notion that diverse microbiota perception signals are integrated into a convergent transcriptional output via various, redundant routes. These observations prompted us to perform a separate differential gene expression analysis using inoculated wild-type plants as a reference to which we compared mutant plant lines under corresponding treatment conditions. While GNSR expression levels in most mutants were similar to those in wild-type plants, they were reduced in *bak1-5* *bkk1* (Extended Data Fig. 7a). Accordingly, elicitors that strongly induced GNSR genes in the analysed datasets^29^ (Extended Data Fig. 6a) and in our experiments (Extended Data Fig. 6b) were shown to require BAK1 and/or BKK1 for signalling (that is, flg22 and elf18)^67^. This suggests that GNSR expression is modulated by BAK1 and/or BKK1 and therefore driven by PTI-dependent processes. Conversely, but less pronounced, *Pseudomonas* Leaf15 caused higher expression levels of several GNSR genes in *rbohD* *rbohF* than in wild-type plants (Extended Data Fig. 7a), suggesting that additional PTI components are involved in GNSR induction. Moreover, axenic *bak1-5* *bkk1* plants also exhibited lower levels of GNSR expression than axenic wild-type plants (Extended Data Fig. 7b; median log_2_FC = −2.55). This indicates that BAK1 and/or BKK1 modulate basal expression levels of GNSR genes even in absence of bacteria—for example, by perceiving endogenous signals (such as phytocytokines like STMP6)^45,68,69^.

In summary, these findings suggest a common regulatory network that orchestrates host reprogramming upon non-self perception. This network involves several redundant PTI components that drive the expression of immunity genes such as GNSR genes, probably through activation by WRKY TFs.

### Leaf microbiota strains modulating immune responses

The finding that boiled culture extracts of leaf microbiota strains (Fig. 4) and synthetic MAMPs^29^ (Extended Data Fig. 6) induced the expression of GNSR genes in *A. thaliana* prompted us to further investigate the link between extracts and MAMPs concerning immune elicitation. To identify bacterial culture extracts with immunomodulatory activity, we screened extracts derived from strains representing all 137 ASVs of the *At*-LSPHERE individually for their ability to trigger ROS bursts in leaves. The latter are produced upon MAMP/DAMP perception by the NADPH oxidase RBOHD, which contributes critically to PTI and microbiota homeostasis^9,50,70,71^.

**Extended Data Fig. 8:**
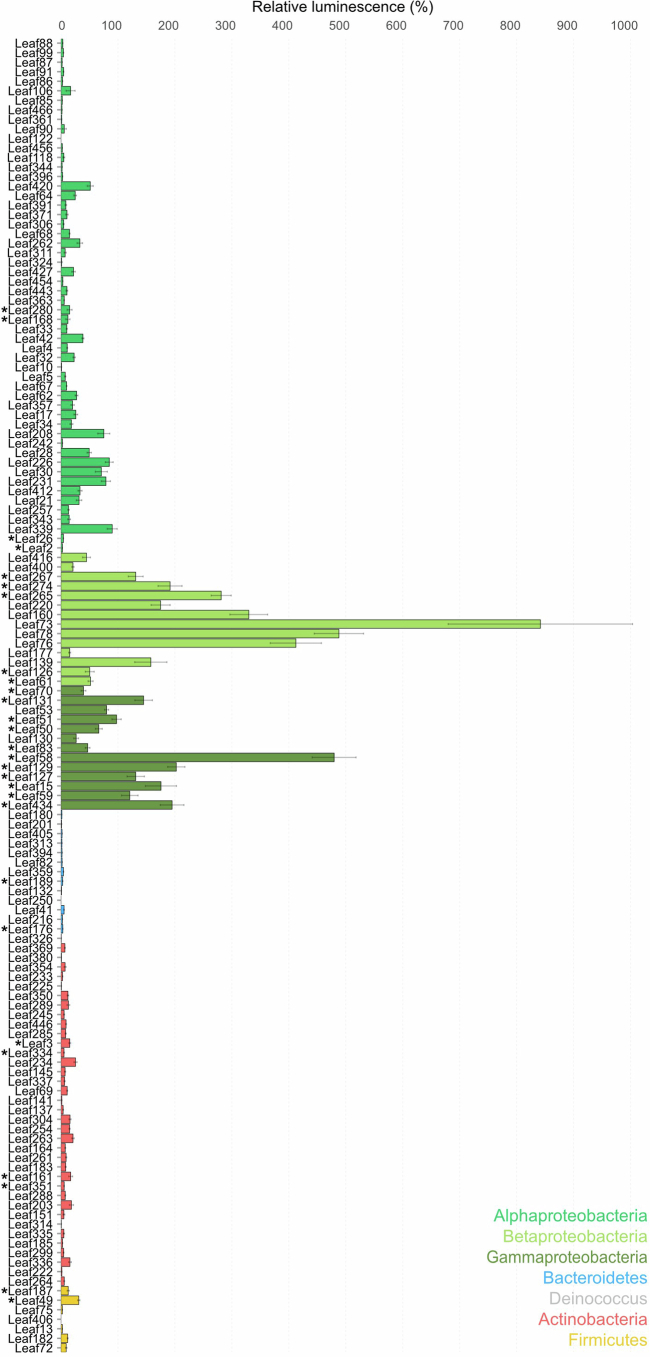
Prevalence of ROS burst elicitation by the leaf microbiota. Total luminescence induced by boiled culture extracts of 137 leaf microbiota strains when applied onto *A. thaliana* Col-0 wild-type leaf disks. Measurements relative to control treatment with 10 nM flg22. Extracts were considered immunogenic if integrated luminescence was greater than that of the extract with highest integrated luminescence that did not exhibit ROS burst dynamics. Data from n = 1 experiment with 8 leaf discs per condition. Strains ordered according to phylogeny, highlighted by color. Columns indicate mean, error bars represent standard error. ROS bursts triggered by extracts of strains marked with an asterisk were previously reported by Pfeilmeier et al. (2021, ref. ^9^) and were included here to cover all strains in the synthetic microbiota SynCom-137 (as shown in Fig. 1, Extended Data Fig. 2). Source data

**Extended Data Fig. 9:**
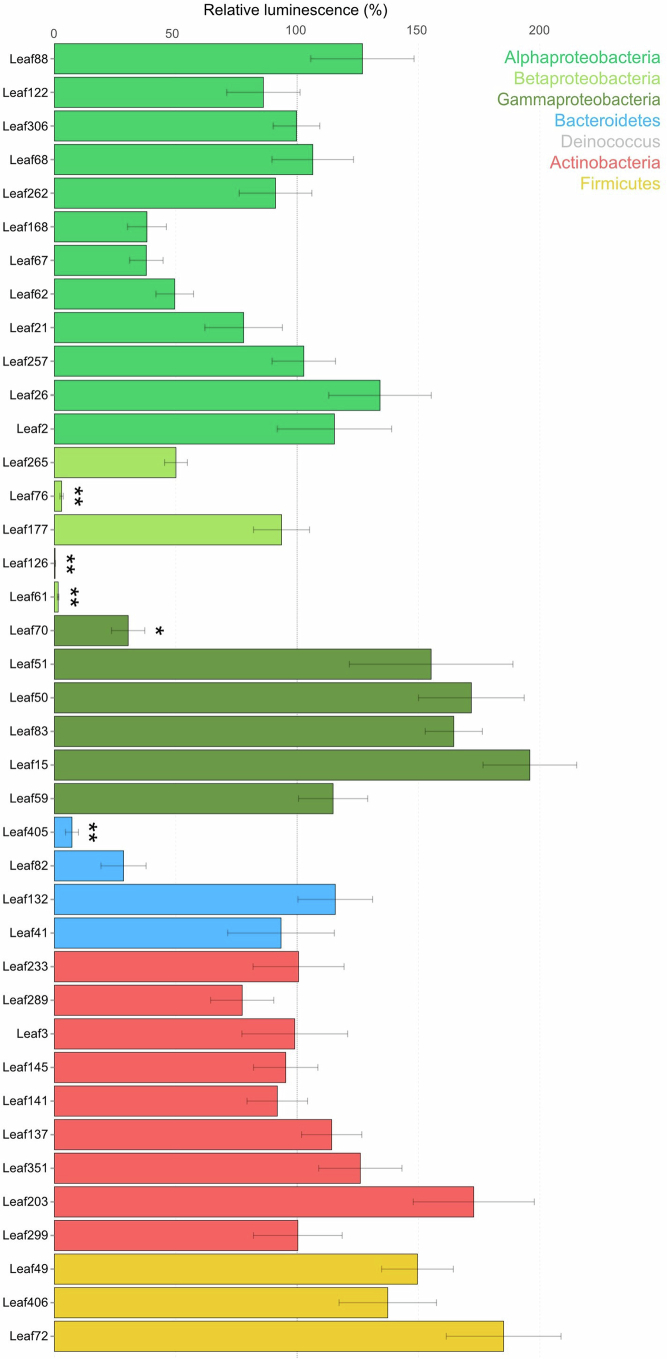
ROS bursts elicited by flg22 in leaf disks pretreated with leaf microbiota strains. ROS bursts triggered by flg22 in leaf disks of *A. thaliana* Col-0 wild-type plants treated with leaf bacteria. Luminescence (x-axis) relative to 100 nM flg22 treatment in mock-pretreated leaf disks, indicated by vertical line at 100%. Strains (y-axis) ordered according to phylogeny, as highlighted by color. Columns indicate mean, error bars represent standard error. Statistical significance was assessed by a two-sided Wilcoxon test with Bonferroni correction relative to mock-pretreatment (**: p ≤ 0.01, and *: p ≤ 0.05). Data from n = 1 experiments each comprising 8 leaf discs per condition. Similar values were observed in an independent experiment (Suppl. Fig. 8). Source data

The screen revealed that 44% of the bacterial extracts induced a ROS burst in *A. thaliana* leaf discs (Extended Data Fig. 8), indicating a high prevalence of potential elicitors from leaf microbiota members. While all extracts derived from Betaproteobacteria and Gammaproteobacteria caused ROS production, none of the Bacteroidetes or Deinococcus-Thermus extracts did. The remaining phyla varied in their fraction of immunogenic extracts (39% of Alphaproteobacteria, 29% of Firmicutes and 24% of Actinobacteria). A subset of 39 strains was chosen to represent varying ROS burst dynamics in leaf discs treated simultaneously with boiled culture extracts and flg22 (Supplementary Fig. 7). This subset contained five strains that, when inoculated onto leaf discs, could significantly suppress subsequent flg22-mediated ROS accumulation (Extended Data Fig. 9 and Supplementary Fig. 8). These included Bacteroidetes, Betaproteobacteria and Gammaproteobacteria, suggesting that various phyla of the leaf microbiota harbour immunomodulatory activity.

**Extended Data Fig. 10:**
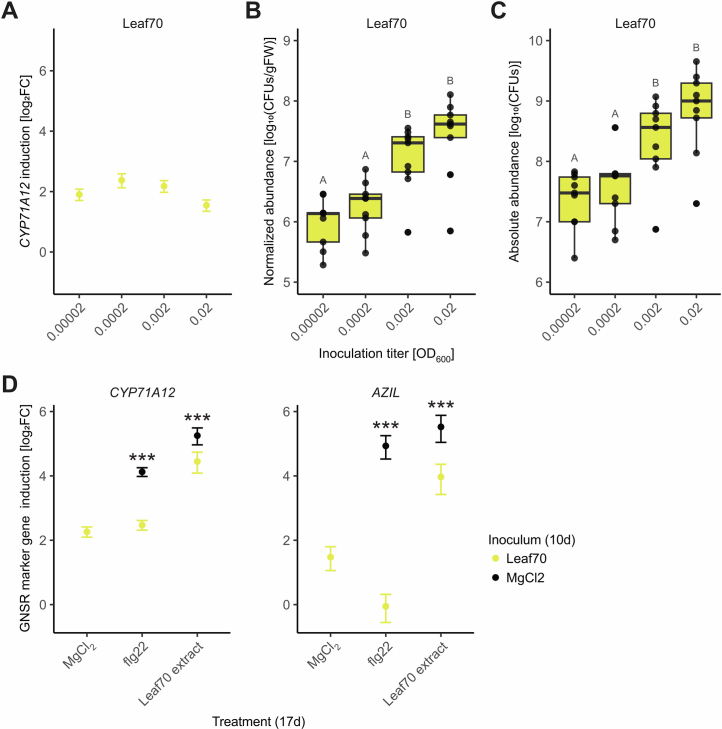
Probing effects of *Stenotrophomonas* Leaf70 on GNSR induction. (**A**) Induction of GNSR gene *CYP71A12* by *Stenotrophomonas* Leaf70 two days after inoculation at indicated density (x-axis), validating weak GNSR induction (one-sample t-test, P-value = 0.002, pooled inoculation titers). Datapoints indicate mean log_2_FCs of normalized gene expression relative to axenic control plants. Error bars indicate standard error. Data from one experiment with n = 1 replicates comprising 18 pooled plants per condition measured in duplicate. (**B**, **C**) Normalized (**B**) and absolute (**C**) abundance of *Stenotrophomonas* Leaf70 *in planta* two days after inoculation titrated by dilution of inoculum density (x-axis). Bars indicate median, hinges indicate first and third quartile, whiskers indicate smallest/largest value within 1.5 × IQR (interquartile range). Letters above points indicate significance levels obtained from one-way ANOVA with Tukey’s post-hoc test. Data from one experiment with n = 9 plants per condition. (**D**) Induction of GNSR genes *CYP71A12* (left) and *AZIL* (right) (y-axis). Plants were inoculated with *Stenotrophomonas* Leaf70 (inoculum OD_600_ of 0.02) or mock-inoculated with MgCl_2_ ten days after sowing. Then, seven days after inoculation, plant responses were triggered with 10 μM flg22, *Stenotrophomonas* Leaf70 extract (prepared from bacterial suspension at an OD_600_ of 2), or mock-triggered with MgCl_2_. Transcriptional response was assessed two days after trigger, indicating that *Stenotrophomonas* Leaf70 can suppress elicitor-triggered GNSR induction. Data points indicate mean log_2_FCs of normalized gene expression relative to axenic control plants. Error bars indicate propagated standard deviation. Statistical significance was assessed by two-sided t-test with Bonferroni correction comparing induction levels between plants inoculated with *Stenotrophomonas* Leaf70 and mock-treated controls (***: p ≤ 0.0001). Data from one experiment with n = 4 replicates comprising a total of 16 plants per condition, measured in duplicate. Source data

Among the tested bacteria was *Stenotrophomonas* Leaf70, which suppressed flg22-mediated ROS burst induction (Extended Data Figs. 8 and 9). This was notable because *Stenotrophomonas* Leaf70 extracts elicited a strong response by themselves (Fig. 4) (although initial cell densities differed). However, the strain was found to cause only a weak transcriptional host response when colonizing the plant^21^ (Fig. 3c,d), a result that we verified by assessing *CYP71A12* induction (Extended Data Fig. 10a–c). In addition to ROS burst inhibition, the divergence in GNSR induction between extracts (Fig. 4) and live bacteria (Fig. 3 and Extended Data Fig. 10a–c) indicates that *Stenotrophomonas* Leaf70 could harbour mechanisms to inhibit plant immune responses that would be triggered by its constituent elicitors, similar to observations of PTI suppression by root commensals^72–74^, mutualists^75,76^ or foliar pathogens^77–81^. To test this hypothesis, we inoculated plants with *Stenotrophomonas* Leaf70, allowed bacteria to establish and the plant to adapt for seven days, and then triggered transcriptional responses in the plant and measured GNSR induction levels after two days. We triggered the GNSR after bacterial establishment with boiled culture extracts of *Stenotrophomonas* Leaf70 to limit elicitors to those potentially present during colonization, as well as with flg22 to link the observed ROS burst suppression to altered gene expression changes in the plant. In mono-association, *Stenotrophomonas* Leaf70 caused the induction of *CYP71A12* and *AZIL* to similar levels as in previous experiments (log_2_FC ≈ 2 relative to the axenic control)^21^ (Extended Data Fig. 10a,d). While treatment with flg22 or highly concentrated boiled culture extracts caused strong induction of the GNSR genes, the presence of live *Stenotrophomonas* Leaf70 diminished induction levels substantially. Strikingly, the induction of *CYP71A12* by flg22 treatment in plants adapted to *Stenotrophomonas* Leaf70 was as strong as in mono-association with the strain (log_2_FC ≈ 2), and the induction of *AZIL* was inhibited (Extended Data Fig. 10d). Considering its potential to impede flg22-mediated ROS production, *Stenotrophomonas* Leaf70 might suppress GNSR induction by inhibiting immune system components that are known to be required for early PTI signalling (including ROS production), such as BAK1/BKK1 or BIK1/PBL1 (ref. ^70^). Indeed, the flagellin-derived flg22 epitope variant of *Stenotrophomonas* Leaf70 is highly immunogenic^19^. We thus hypothesize that *Stenotrophomonas* Leaf70 might suppress GNSR induction to inhibit the expression of immunity genes that would reduce its capacity to proliferate.

## Discussion

Our investigation of the GNSR in the context of microbiota assembly establishes new roles of *IGMT3*, *CHX16*, *CYP71A12*/*CYP71A13* and *MLO12* in the assembly of the plant microbiota (Fig. 1), supporting the importance of plant immunity in this process^8–10^. Taxa consistently enriched in the microbiota of these mutant plants tended to induce a strong GNSR (Extended Data Figs. 3 and 4a). These findings suggest that the GNSR is involved in a feedback system that modulates phyllosphere colonization upon response induction. In fact, we found that the GNSR components involved in microbiota assembly directly affected the colonization ability of a commensal that induces a strong host response (*Arthrobacter* Leaf137), a foliar pathogen (Pst) and a microbiota strain that is considered commensal but exhibited context-dependent pathogenicity (*Serratia* Leaf50) (Fig. 2 and Extended Data Fig. 4b). This observation suggests that the GNSR acts on diverse bacteria and contributes to a balanced assembly of the microbiota by modulating leaf colonization by individual strains. Along with the robust response induction in immunocompromised plants (Fig. 5c and Supplementary Fig. 6) and the increased susceptibility of GNSR mutants to pathogen infection^21^, this underscores the role of a convergent non-self response in sustaining plant health, which depends on both the microbiota and functional immunity^9,11,13,28,82^. We have also shown that various microbiota members modulated MAMP-triggered ROS production in leaves (Extended Data Fig. 9) and as an example highlighted *Stenotrophomonas* Leaf70, which can suppress the induction of GNSR genes (Extended Data Fig. 10), suggesting that certain leaf bacteria may benefit from the inhibition of immunity genes in the phyllosphere. However, the mechanism and spatial range of suppression of these responses (particularly by *Stenotrophomonas* Leaf70), as well as a causal effect on bacterial abundance, remain to be demonstrated. Similar to our observation, the ability to suppress immunity genes was found in about 40% of root microbiota strains—for example, through the secretion of acids that modulate environmental pH or effectors that alter elicitor immunogenicity^72–74,81^. Considering these observations and the predictive nature of the GNSR in terms of transcriptional reprogramming, we hypothesize a continuous function of dose-responsive regulation of immune responses to bacteria of various lifestyles, rather than a differentiation between pathogens and commensals per se.

While strong immune responses elicited by pathogen-derived MAMPs and their suppression through the secretion of effectors are well studied^72–80^, our data show an important overlap in plant responses, particularly involving host genes involved in tryptophan-derived secondary metabolism, which are functional regarding pathogens and microbiota strains by affecting bacterial abundances in planta. Indeed, a relationship between bacterial abundance and the plant transcriptional response was previously implied, as strong response elicitors tended to colonize leaves more extensively^21^ (Supplementary Fig. 9a). Here we examined this relationship in detail for several microbiota members. Our data reveal that microbiota strains that differ in their leaf colonization ability cause dynamic transcriptional responses varying in magnitude and timing (Fig. 3 and Extended Data Fig. 5). Together with our examination of responses to bacterial extracts and purified MAMPs (Fig. 4 and Extended Data Fig. 10), we conclude that these differences in host response are collectively driven by bacterial abundance, exposure time and the molecular composition of bacterial cells. The integration of these factors then results in the convergent, dose-responsive expression of immunity genes. Plant–microbiota interactions are thus critically shaped by a strain’s ability to colonize plant tissue. We exemplarily analysed carbon versatility as a potential factor providing an advantage during colonization^83^ and found that the ability of microbiota strains to use more carbon sources for growth correlated significantly with their potential to induce plant responses (Supplementary Fig. 9b). Together, these observations suggest a relationship between the degree of exposure to individual strains and plant response intensity.

We provide evidence that the GNSR is a robust plant immune response, as various signalling pathways seem to redundantly trigger its induction (Fig. 5c and Supplementary Fig. 6). This is intriguing in light of the proposition that PRRs are subject to selective pressures that favour diversification of signal perception rather than downstream signal propagation^84^, underscoring a central role of a convergent signalling system in immune response regulation. Importantly, we unify previously distinct responses in PTI signalling with the GNSR as a congruent non-self response system that relies on a redundant regulatory network, where WRKY TFs are predicted to be involved (Fig. 5a, Extended Data Fig. 6a and Supplementary Fig. 2). While we could determine that canonical immunity components such as BAK1/BKK1 and RBOHD/RBOHF modulate GNSR expression (Fig. 5c and Extended Data Fig. 7), it is remarkable that this immune response was consistently induced in higher-order mutants such as *min7* *bak1-5* *bkk1* *cerk1* and *min7* *fls2* *efr1* *cerk1* (Supplementary Fig. 6), which are severely compromised in elicitor perception and immune signalling^8,62–66^. However, the prolonged exposure to elicitors during bacterial colonization in these experiments could mask the specific contributions of individual signalling pathways to GNSR induction (Fig. 5c and Supplementary Fig. 6), requiring examinations with higher temporal resolution of the host response. Remaining canonical PTI signalling pathways that are unimpaired by deficiencies in the tested mutants include, for example, the perception of bacterial hydroxylated fatty acids (such as 3-OH-FA) by the PRR LORE^85^. However, hydroxylated fatty acids are generally weak PTI inducers^29^, rendering them unlikely to be the sole elicitors that cause extensive GNSR induction. Recent evidence suggests that PRR-independent pathways can induce PTI responses through the action of bacterial toxins^86^, which we cannot exclude as inducers of the GNSR in our dataset and which could be integrated as additional microbiota signals along with conserved patterns to regulate immune responses. Future studies aimed at identifying signalling components that collectively cause the induction of immunity genes, including the GNSR, will thus require the generation and testing of broad higher-order mutant plant lines. However, whether such increasingly immune-deficient plants are viable remains elusive, particularly considering safeguard mechanisms that trigger autoimmunity upon the perturbation of central PTI components (for example, as recently described for BAK1/BKK1 (ref. ^87^)). In summary, our work opens intriguing perspectives on potentially uncharacterized upstream signalling events that contribute to a convergent core immune response that impacts plant–microbiota interactions.

## Methods

### Plant growth conditions

For gnotobiotic assays, *A. thaliana* seeds were sterilized as described previously^88^ and stratified for four days in the dark at 4 °C before sowing. The plant lines used in this study with sources and references can be found in Supplementary Table 5.

Plants used to profile microbiota composition on GNSR mutants were grown in a gnotobiotic system based on calcined clay (Calcined Clay Drying Agent, Diamond Pro) supplemented with 0.5× Murashige and Skoog (MS) medium including vitamins (M0222.0050, Duchefa) set to a pH of 5.8, in round microboxes (no. O118/80+OD118 with green filter lid, Sac 02) as described previously^30^. Growth chambers (CU-41L4, Percival) were fitted with full-spectrum lights (Master TL-D 18 W/950 Graphica, Philips) and UVA/UVB lights (Reptistar F18 W/6500 K, Sylvania). The plants were subjected to an 11-h light cycle, with adjusted irradiation intensities of 220 µmol m^−2^ s^−1^ and 5.4 µmol m^−2^ s^−1^ for full-spectrum and UV lights, respectively. The temperature was set to 22 °C and relative humidity to 54%. Twenty seeds of the same genotype were sown per microbox. The day before inoculation, excess seedlings were removed with sterile tweezers to reduce the numbers to five plants per microbox, and each plant was watered with 200 µl of growth medium. Watering was repeated 1.5 and 3 weeks after inoculation. Plants to monitor population sizes on the GNSR mutants were grown in the same substrate in six-well tissue culture plates (92006, TechnoPlasticProducts), as described previously^83^.

Plants grown to assess transcriptional responses were cultivated in a gnotobiotic system based on MS medium including vitamins (M0222.0050, Duchefa) set to a pH of 5.8 and supplemented with 3% w/v sucrose (84100, Sigma-Aldrich) and 0.55% w/v agar (P1001.1000, Duchefa), in 24-well plates (92024, TPP Techno Plastic Products) in growth chambers (CU-41L4, Percival) equipped with full-spectrum lights (Alto II 17 W/841, Philips). For the first 14 days of incubation, the plates were sealed with parafilm (PM-996, Bemis). The plants were subjected to a 16-h light cycle for the first week and then to a 9-h light cycle until harvest, with an adjusted irradiation intensity of 220 µmol m^−2^ s^−1^. The temperature was set to 24 °C during light periods and 22 °C during dark periods, at a relative humidity of 65%.

For the ROS burst assays, *A. thaliana* plants were grown in potting soil (Substrate 1, Klasmann-Deilmann) for five to six weeks under an 11-h light cycle in growth chambers (CU-41L4, Percival) fitted with full-spectrum lights (Master TL-D, 18 W/840, Philips) and set to 22 °C and 60% relative humidity.

Plants used for the fluorometric reporter gene assay were cultivated hydroponically in 96-well microplates (92026, TPP Techno Plastic Products) in 0.5× MS basal salt medium without vitamins (M0221.0025, Duchefa) supplemented with 0.5% sucrose and adjusted to pH 5.8. The plants were subjected to a 16-h light cycle using full-spectrum lights (Alto II 17 W/841, Philips) with an adjusted irradiation intensity of 52 µmol m^−2^ s^−1^ in growth chambers (CU 41L4, Percival). The temperature was set to 22 °C and the relative humidity to 65%. The lids of the 96-well plates were sealed with parafilm during the incubation of the plants.

### Bacterial cultivation conditions, inoculation of seedlings and treatment with elicitors

Strains from the *At*-LSPHERE collection^37^ were grown on R-2A agar (17209, Sigma-Aldrich) supplemented with 0.5% v/v methanol (R2A + M) (32213, Sigma-Aldrich) at room temperature (approximately 22 °C). For the inoculation of plants, bacteria were recovered from agar and resuspended in 10 mM MgCl_2_ (63068, Sigma-Aldrich) by vortexing for 5 min and adjusted to an OD_600_ of 0.2. Inoculation suspensions were prepared by as many tenfold dilution steps as required for the experiment. Seedlings were inoculated ten days after sowing with 10 µl of bacterial suspension, which was equally distributed onto all leaves and the centre of the rosette. Axenic control plants were analogously mock-inoculated with 10 mM MgCl_2_. For dose–response experiments with live bacteria or boiled culture extracts, seedlings were inoculated/treated 17 days after sowing.

Treatments with purified elicitors flg22 (GenScript), elf18 (EZBiolab), 3-OH-FA (provided by S. Ranf) or STMP6 (GenScript) were performed analogously to the inoculation of seedlings with bacteria as described above, 17 days after sowing.

For the fluorometric reporter gene assay, plants were treated 12 days after sowing with 10× concentrated bacterial suspensions (based on OD_600_) that were diluted to the desired final concentration in the plant growth medium. Axenic control plants were mock-inoculated with the corresponding volume of 0.5× MS medium.

The synthetic microbiota (SynCom-137) contained a single strain for each ASV of the *At*-LSPHERE^37^ (Supplementary Table 1) and had been mixed as described previously^9^. Several frozen glycerol stocks of the suspended SynCom-137 were thawed at 25 °C for 3 min, subjected to centrifugation at 11,000 *g* for 10 min and washed twice by centrifugation at 11,000 *g* for 2 min. The pellets were resuspended and pooled in 10 mM MgCl_2_, and OD_600_ was adjusted to 0.02. Seedlings were inoculated analogously to inoculation with single strains. Four aliquots were separated as controls to determine the bacterial composition of the inoculum. The presence of all ASVs in the inoculum was validated with a few exceptions, probably due to insufficient sequencing depth (Supplementary Table 6).

### Harvest of plant material

To enumerate bacteria, phyllosphere samples were obtained by removing cotyledons and the rhizosphere from seedlings using sterilized scalpels and forceps. The samples were transferred into individual plastic tubes (72.695.500, Sarstedt) containing 200 µl of 10 mM MgCl_2_ and a stainless-steel bead (KU.5 NIRO 403, Berani Kugellager). After recording the sample fresh weights, the plant material was homogenized by bead beating (TissueLyzer II, Qiagen) at 30 Hz for 45 s and subjected to tenfold serial dilution. The dilution series was plated on R2A + M agar to determine colony-forming units and verify gnotobiotic conditions.

Samples to assess transcriptional plant responses were collected 19 days after sowing by isolating the phyllosphere as described above. The samples were then flash-frozen in liquid nitrogen and transferred into screw-cap tubes (60.558.001, Sarstedt), pooling four to six samples of each technical replicate of the same condition, and stored at −80 °C. Up to nine remaining seedlings were harvested individually to enumerate bacteria as described above. Where gnotobiotic conditions were confirmed, all samples of each technical replicate were combined to one sample of 12–18 pooled plants. Plant RNA was prepared using the Quick-RNA Plant Kit (R2024, Zymo) according to the manufacturer’s instructions (including on-column DNA digestion), and concentration and purity were assessed with a spectrophotometer (ND-1000, NanoDrop).

To generate samples for microbiota profiling, plants were harvested 3.5 weeks after inoculation, and phyllosphere samples were obtained as described above. From six microboxes per condition, three samples of each microbox were transferred into individual screw-cap tubes for DNA extraction using the FastDNA SPIN Kit for Soil (116560200, MP Biomedicals). The samples were lyophilized at −40 °C and 0.12 mbar for 16 h (Alpha 2-4 LD Plus, Christ) and homogenized twice at 30 Hz for 45 s (TissueLyzer II, Qiagen), and DNA was prepared according to the kit manufacturer’s instructions.

### Fluorescence-based gene expression analysis in *pCYP71A12*::*GUS* reporter line seedlings

β-Glucuronidase activity in intact *pCYP71A12*::*GUS* reporter line seedlings was quantified in a fluorometric assay as described previously^89^. Briefly, the plant growth medium was removed; replaced with assay solution containing 50 mM sodium phosphate pH 7.0, 10 mM EDTA (03677, Sigma-Aldrich), 0.1% Triton X-100 (T9284, Sigma-Aldrich) and 1 mM 4-MUG (B21190.MD, Thermo Fisher Scientific); and incubated for 6 h at 37 °C in the dark. The enzymatic assay was stopped by the addition of 250 mM Na_2_CO_3_, after which the reagent mix was transferred to an opaque plate to determine 4-MU product fluorescence in a plate reader (BioTek Synergy H1, Agilent) using excitation and emission wavelengths of 365 nm and 455 nm, respectively.

### RNA sequencing and differential gene expression analysis

Poly-A enriched mRNA libraries were prepared and sequenced on an NovaSeq 6000 (Illumina) using paired-end sequencing (2 × 150 bp). Library preparation and sequencing was performed by Novogene (https://www.novogene.com) and on average generated 20 million reads per sample. The resulting raw reads were cleaned by removal of adaptor sequences, low-quality-end trimming and removal of low-quality reads using BBTools v.38.18 (ref. ^90^). The exact commands used for quality control can be found on the Methods in Microbiomics webpage (https://methods-in-microbiomics.readthedocs.io)^91^. Transcript abundances were quantified using Salmon v.1.10.1 (ref. ^92^) and TAIR10 (ref. ^93^). Differential gene expression analysis was performed using DESeq2 v.1.37.4 (ref. ^53^). Data analysis and visualization were performed in RStudio Server (v.2022.7.0.548, RStudio Team).

### RT-qPCR and expression analysis

The concentrations of all RNA samples within each experiment were adjusted to 100 or 200 ng µl^−1^. Complementary DNA was synthesized on a Biometra TRIO 48 (846-2-070-723, Analytik Jena) using the RT^2^ HT First Strand kit (330411, Qiagen), according to the manufacturer’s instructions. The samples were diluted with ddH_2_O to obtain enough volume for the required number of amplification reactions and subjected to RT-qPCR using FastStart Universal SYBR Green Master (4913914001, Roche) on a QuantStudio 7 Flex Real-Time PCR System (4485701, Applied Biosystems), according to the manufacturers’ instructions. Each reaction was performed in technical quadruplicates or duplicates. The PCR comprised an initial denaturation (95 °C, 10 min), followed by 40 cycles of denaturation (95 °C, 15 s) and elongation (60 °C, 60 s), and a melting curve (60–95 °C, 0.05 °C s^−1^) with final dissociation (95°, 15 s). *CYP71A12* was amplified using the primers CYP71A12_LP (5′-TGACAGTGGCCAACCTTGTAGG) and CYP71A12_RP (5′-TGCAATGAGAGGGAACTTTCGG). *AZIL* was amplified using the primers AZIL_LP (5′-ACCACTGCTACTGATTGTCGATGC) and AZIL_RP (5′-TAGGACTCGGGACCTTTGGACTTG). The housekeeping gene *ACT2* (ref. ^94^) was amplified using the primers ACT2_LP (5′-TCCCTCAGCACATTCCAGCAGAT) and ACT2_RP (5′-AACGATTCCTGGACCTGCCTCATC) and was subsequently used as a reference gene for data normalization. Linear amplification was verified for each target gene in each RT-qPCR experiment with a dilution series of the sample with the highest expected gene expression. The raw data were analysed and processed using the proprietary software QuantStudio Real-Time PCR System Version 1.3 (Applied Biosystems) and subsequently analysed and visualized in RStudio Server (v.2022.7.0.548, RStudio Team). Fold changes of the normalized expression of target genes in test conditions were determined relative to axenic control plants as described previously^95^.

### Bacterial 16S rRNA gene sequencing

The DNA concentration of all samples was assessed using dsDNA QuantiFluor (E2670, Promega) and normalized to 1 ng µl^−1^. A 16S rDNA amplicon library was prepared as described previously^11^. The first PCR was performed using the primers 799F^96^ and 1193R^97^ and DFS-Taq polymerase (101100, Bioron). Aliquots of each product were analysed by agarose gel (2%) electrophoresis to validate the presence of amplicons (or their absence in control samples). To remove excess primers, the amplification products were subjected to clean-up using Antarctic phosphatase (M0289, New England Biolabs) and Exonuclease I (M0293, New England Biolabs), according to the manufacturer’s instructions. A second PCR to amplify 16S rDNA amplicons with barcoded primers was performed as described previously^9,37^. Aliquots of each amplification product were analysed by agarose gel (2%) electrophoresis to estimate product quantities. Samples from each PCR run were pooled at approximately equal quantities and subjected to AMPure XP bead-based clean-up (A63881, Beckman Coulter). To separate bacterial from plastid 16S rDNA, the samples were analysed by agarose gel (2%) electrophoresis and purified from gel using the NucleoSpin clean-up kit (740609, Macherey-Nagel). All pools were combined after DNA concentration was measured with QuantiFluor (E2670, Promega) to achieve equal sample volume ratios in the library. The pooled library was subjected to AMPure XP bead-based clean-up (A63881, Beckman Coulter) twice at a bead–DNA ratio of 0.7. The final DNA library was denatured, diluted and spiked with PhiX (10%), according to the manufacturer’s instructions for sequencing using the MiSeq Reagent Kit v.3 (600-cycle) (2 × 300 bp paired-end, MS-102-3003, Illumina), performed at the Genetic Diversity Centre Zurich with custom sequencing primers, as described previously^37^.

### Processing of 16S amplicon sequencing data and analysis

Reference 16S rRNA gene sequences for ASVs in SynCom-137 were obtained as described previously^30^. Reads from paired-end DNA sequencing were merged and processed using USEARCH v.11.0.667-i86 linux64 (ref. ^98^) (which includes the UPARSE algorithm^99^), and ASV count tables were analysed in RStudio Server (2022.7.0.548, RStudio Team), as described previously^9^.

### Preparation of bacterial extracts

Bacterial extracts to assess transcriptional plant responses were prepared by resuspending bacteria grown on R2A + M agar in 10 mM MgCl_2_ and adjusting the suspensions (as described above) to an OD_600_ of 2. The suspensions were boiled for 30 min at 100 °C while being gently shaken. Boiled culture extracts were then diluted to the required density (relative to OD_600_ before boiling). After dilution, each boiled culture extract was subjected to tenfold serial dilution and plating on R2A + M agar to validate the complete inactivation of the bacteria.

Extracts for the ROS burst assays were prepared from bacterial suspensions adjusted to an OD_600_ of 10 in ddH_2_O. The suspensions were then boiled at 100 °C for 10 min with intermediate vortexing, subjected to sonication in a water bath (2210E-MT, Branson Ultrasonics) for 5 min and cooled on ice. Extracts were obtained by collecting the supernatant after centrifugation for 7 min at 16,000 *g* and 4 °C. The extracts were stored at −20 °C.

### Measuring ROS production in leaves

Luminol-based ROS burst assays were performed as described previously^9,67^. Briefly, eight leaf discs (4 mm in diameter) per treatment condition were placed into individual wells of a 96-well plate (655075, Greiner Bio-One) filled with 100 µl ddH_2_O and incubated overnight in the dark at room temperature (approximately 22 °C). Then, the ddH_2_O was removed, and 100 µl of a treatment solution containing 17 μg ml^−1^ luminol (123072, Sigma-Aldrich) and 10 µg ml^−1^ horseradish peroxidase (P6782, Sigma-Aldrich) was added. Luminescence upon treatment was measured using a plate reader (Victor3, Perkin Elmer) by recording photon counts at an exposure time of 0.5 ms in intervals of 90 s for 60 min.

To assess interference between bacterial extracts and flg22 in ROS burst induction, the treatment solution was additionally supplemented with a combination of either bacterial extract (1:10 dilution, at a final equivalent of OD_600_ of 1 before boiling) and 10 nM flg22 (RP19986, GenScript) or sterile deionized H_2_O and 10 nM flg22. To normalize ROS burst measurements between experiments, the integrated area under the curve of the measured samples was divided by the area under the curve of treatment with 10 nM flg22.

To determine the modulation of flg22-induced ROS bursts by live bacteria, leaf discs were incubated overnight as described above, but in 100 µl ddH_2_O supplemented with 20 µl of bacterial suspensions at an OD_600_ of 0.12 (in 10 mM MgCl_2_, resuspended from agar as described above) to reach a final OD_600_ of 0.02. Mock pretreatments consisted of replacing bacteria with 20 µl of 10 mM MgCl_2_. The bacterial suspension was then replaced by the same treatment solution as described above supplemented with 100 nM flg22.

### Data analysis and visualization

If not otherwise stated, data was analysed and visualized using RStudio Server (2022.7.0.548, RStudio Team) running R version 4.2.1.

### Reporting summary

Further information on research design is available in the Nature Portfolio Reporting Summary linked to this article.

## Supplementary information

Supplementary Information Supplementary Figs. 1–9.

Reporting Summary

Supplementary Table 1 List of *At*-LSPHERE strains in the synthetic microbiota SynCom-137.

Supplementary Table 2 Regulatory motifs obtained from ISMARA using RNA sequencing data of transcriptional plant responses to leaf bacteria.

Supplementary Table 3 Predicted target genes obtained from ISMARA using RNA sequencing data of transcriptional plant responses to leaf bacteria.

Supplementary Table 4 Regulatory motifs obtained from ISMARA using RNA sequencing data of transcriptional plant responses to synthetic MAMPs and DAMPs.

Supplementary Table 5 List of all plant lines used in this study.

Supplementary Table 6 Counts of ASVs obtained from microbiota profiling of GNSR mutants by bacterial 16S rRNA gene sequencing and corresponding metadata.

## Source data

Source Data Fig. 1 Statistical source data.

Source Data Fig. 2 Statistical source data.

Source Data Fig. 3 Statistical source data.

Source Data Fig. 4 Statistical source data.

Source Data Fig. 5 Statistical source data.

Source Data Extended Data Fig. 1 Statistical source data.

Source Data Extended Data Fig. 2 Statistical source data.

Source Data Extended Data Fig. 3 Statistical source data.

Source Data Extended Data Fig. 4 Statistical source data.

Source Data Extended Data Fig. 5 Statistical source data.

Source Data Extended Data Fig. 6 Statistical source data.

Source Data Extended Data Fig. 7 Statistical source data.

Source Data Extended Data Fig. 8 Statistical source data.

Source Data Extended Data Fig. 9 Statistical source data.

Source Data Extended Data Fig. 10 Statistical source data.

## Data Availability

The RNA sequencing data can be found in the European Nucleotide Archive under accession number PRJEB67453 (ERP152478). The DNA sequencing data can be found in the European Nucleotide Archive under accession number PRJEB80640 (ERP164609). Source data are provided with this paper.
