# Supplementary material for: Plant microbiota feedbacks through dose-responsive expression of general non-self response genes
Source: Nat Plants. 2024 Dec 3;11(1):74–89. doi: 10.1038/s41477-024-01856-z (PMC11757152; doi:10.1038/s41477-024-01856-z)
Supplement: Supplementary file 1 — Supplementary Figs. 1–9. [file 41477_2024_1856_MOESM1_ESM.pdf]

nature plants

Supplementary information

https://doi.org/10.1038/s41477-024-01856-z

Plant microbiota feedbacks through dose-responsive expression of general non-self response genes

In the format provided by the authors and unedited

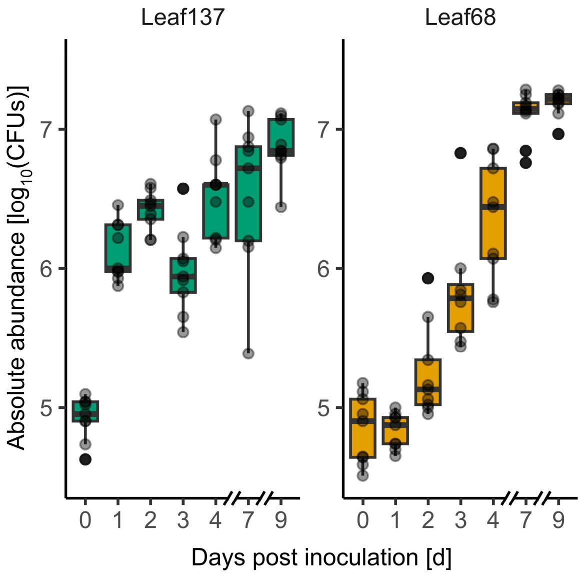

Suppl. Figure 1: Absolute populations of *Arthrobacter* Leaf137 and *Rhizobium* Leaf68 during colonization. Absolute abundances (y-axis) of indicated leaf microbiota strains during nine days of colonization (x-axis). Bars indicate median, hinges indicate first and third quartile, whiskers indicate smallest/largest value within 1.5 × IQR (interquartile range). Data from one experiment with n=9 plants per condition.

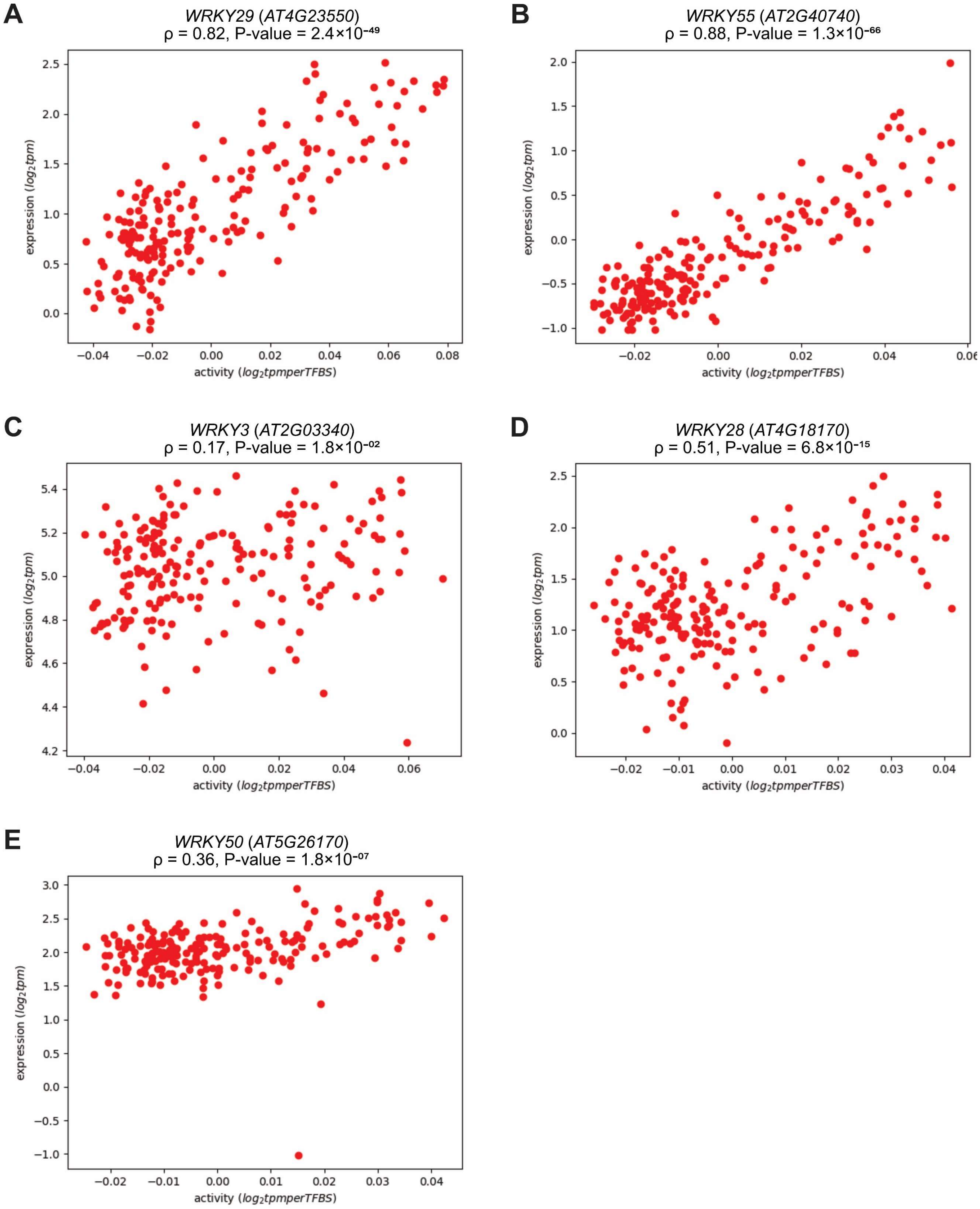

Suppl. Figure 2: WRKY transcription factors (TFs) predicted to drive host reprogramming upon microbiota perception. (A-E) Correlation analyses between inferred activity of regulatory motifs highlighted in Figure 5A (x-axis) and measured expression level of the corresponding WRKY TF (y-axis), as determined by ISMARA, indicating that the TFs are positive regulators of gene expression. Indicated on top of each plot are TF names and gene loci, Pearson correlation coefficient (ρ), and P-value.

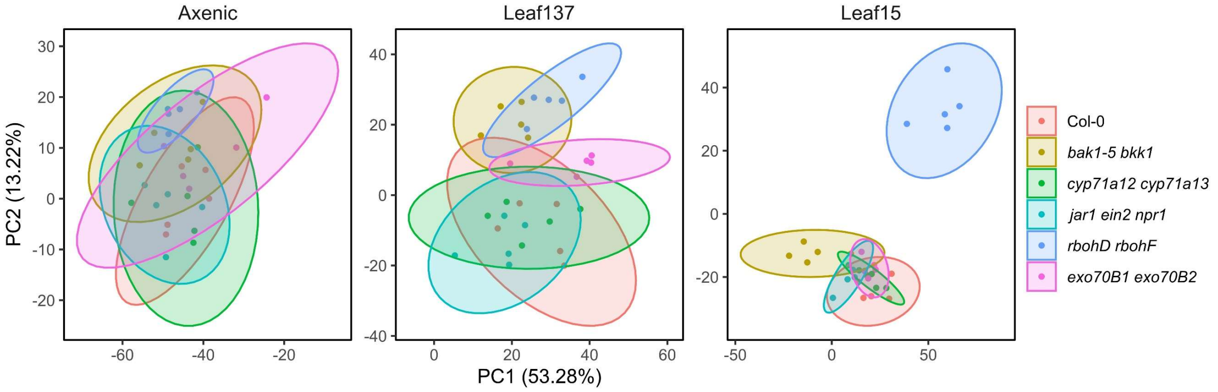

Suppl. Figure 3: Transcriptional response to *Arthrobacter* Leaf137 and *Pseudomonas* Leaf15 in immunity and signaling mutants. Principal component analysis (PCA) of differentially expressed genes (P-value ≤ 0.01, two-sided Wald test with Benjamini-Hochberg correction, filtered for |$\log_2$FC ≥ 1| relative to axenic control in at least one condition) across genotypes (indicated by color) within treatment conditions (indicated on top of each plot), showing distinct responses to microbiota strains in *bak1-5 bkk1* and *rbohD rbohF*. Data from n=5 independent experiments, each comprising 18 pooled plants per condition, sampled nine days post inoculation.

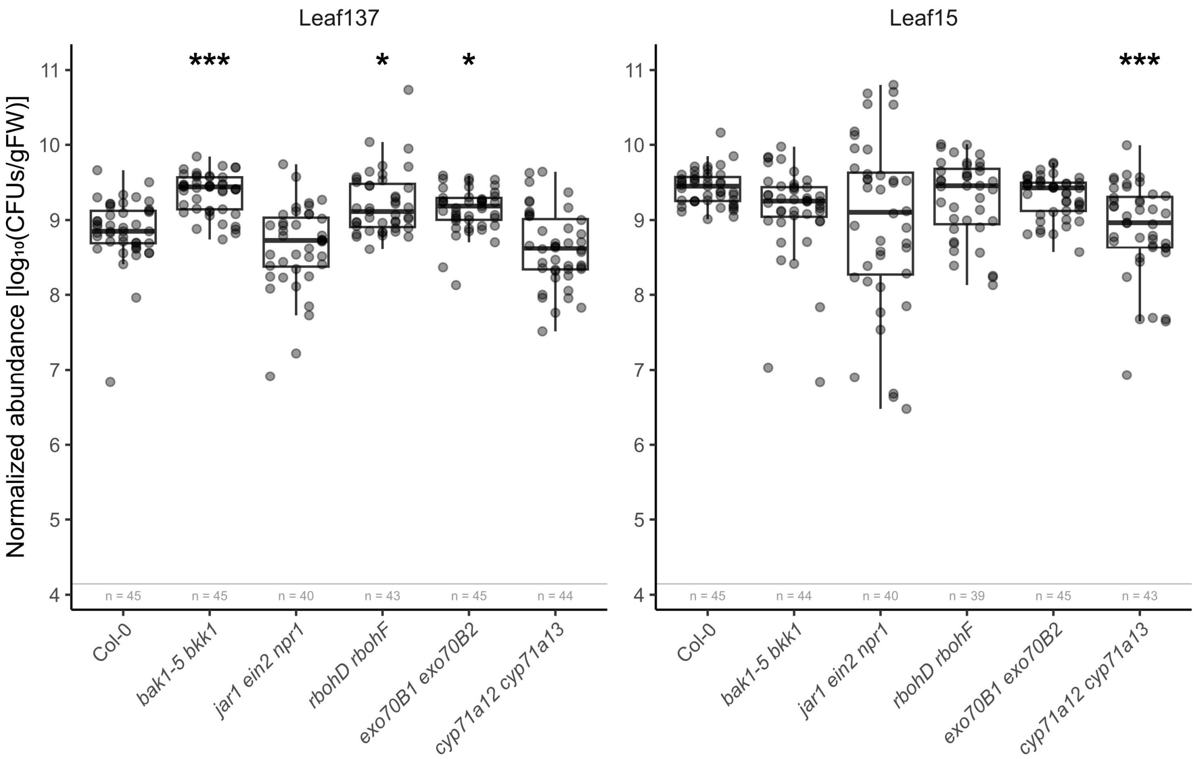

Suppl. Figure 4: Colonization of immunity and signaling mutants by *Arthrobacter* Leaf137 and *Pseudomonas* Leaf15. Bacterial abundances (y-axis) of microbiota strains (indicated on top of plots) in *A. thaliana* Col-0 wild-type and mutant plant lines (x-axis) nine days after inocualtion. Bars indicate median, hinges indicate first and third quartile, whiskers indicate smallest/largest value within 1.5 × IQR (interquartile range). Data from five independent experiments with n=39–45 plants per condition in total (indicated above x-axis). The horizontal line indicates the approximate detection limit. Statistical significance was determined by two-sided t-test with Bonferroni correction using "Col-0" as reference group (***: $p \leq 0.0001$, **: $p \leq 0.001$, and *: $p \leq 0.01$).

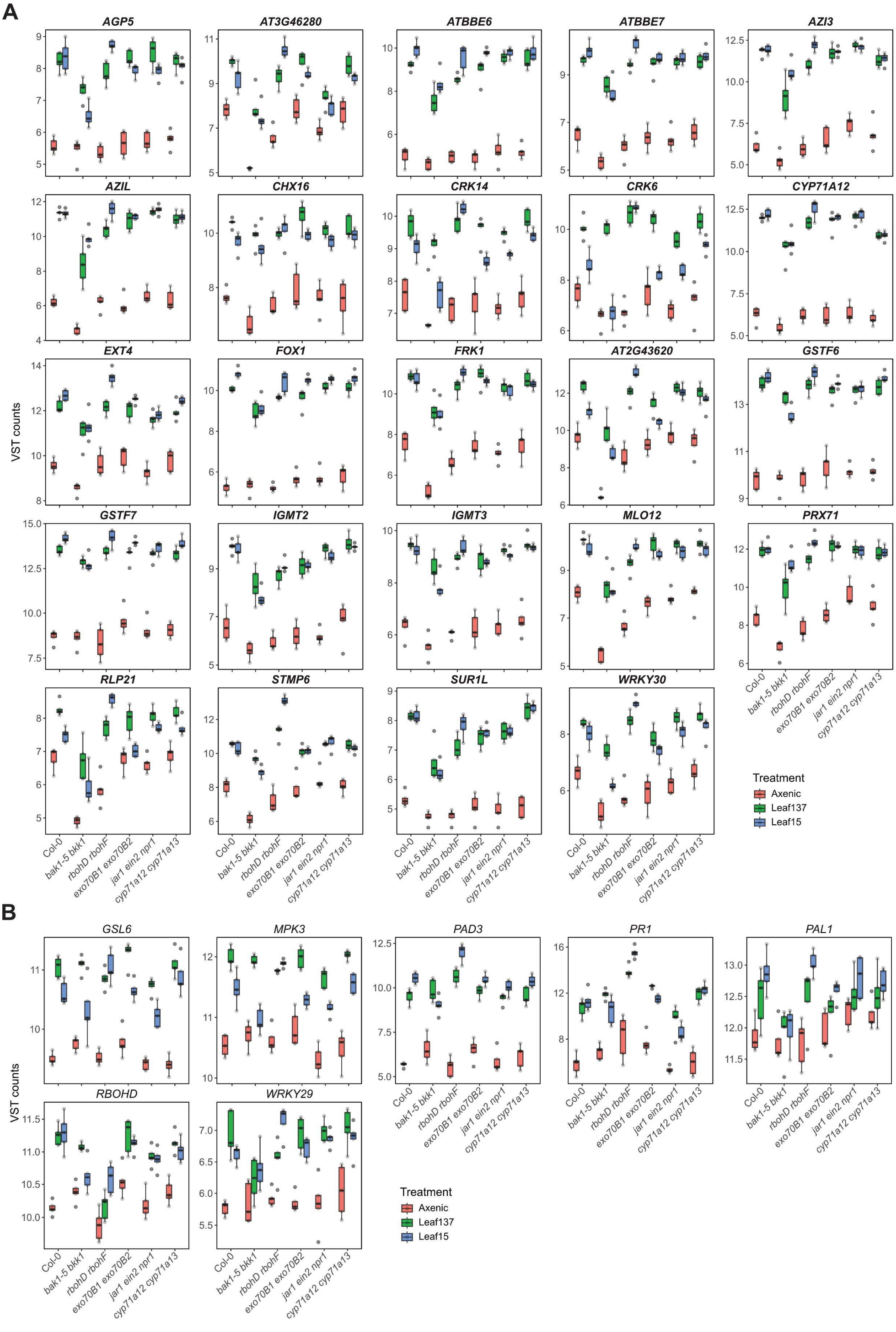

Suppl. Figure 5: GNSR and PTI expression in immunity and signaling mutants. Expression levels (y-axis, variance-stabilizing transformation (VST) counts) of GNSR genes (A) and selected PTI marker genes (B) (indicated above plots) in *A. thaliana* Col-0 wild-type and mutant lines compromised in immunity signaling (x-axis), in presence or absence of *Arthrobacter* Leaf137 or *Pseudomonas* Leaf15 (indicated by color). Bars indicate median, hinges indicate first and third quartile, whiskers indicate smallest/largest value within 1.5 × IQR (interquartile range). (A–B) Data from n=5 independent experiments, each comprising 18 pooled plants per condition, sampled nine days post inoculation.

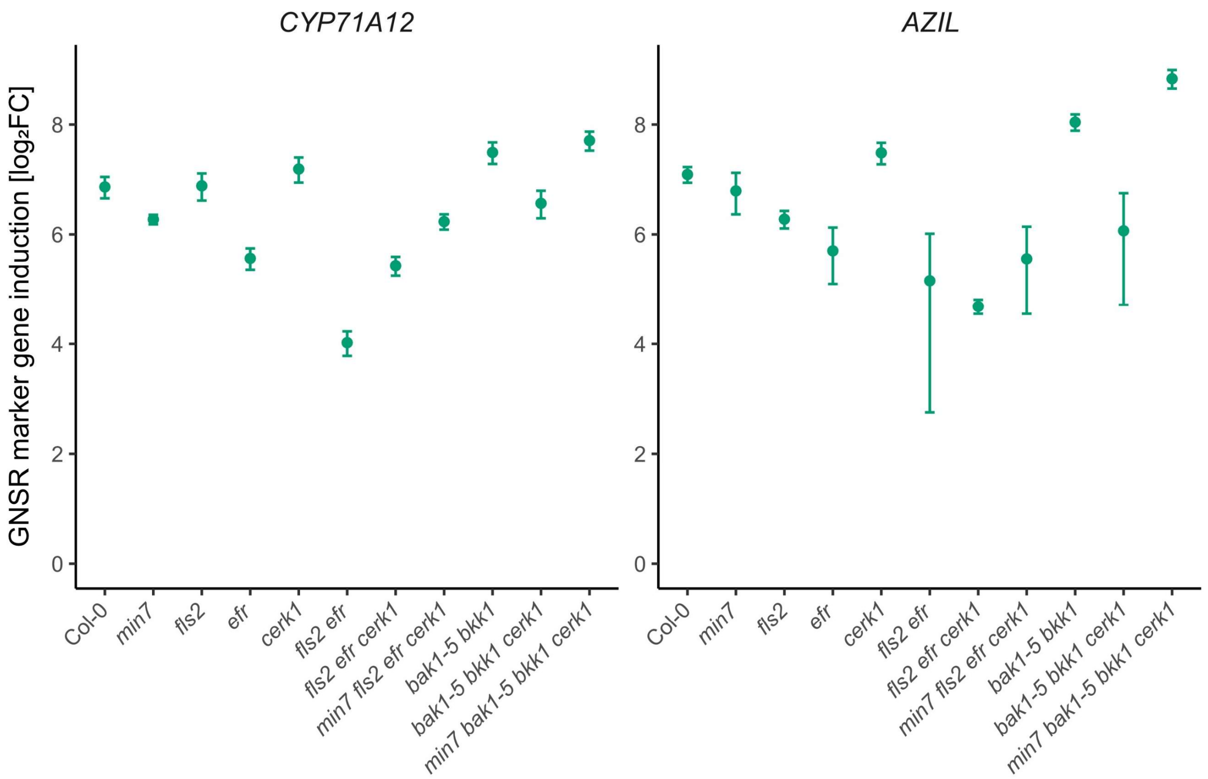

Suppl. Figure 6: GNSR in mutant plants with broad deficiencies in PTI signaling. Induction of GNSR genes *CYP71A12* (left) and *AZIL* (right) nine days after inoculation with *Arthrobacter* Leaf137, showing induction of GNSR in plants lacking key PTI signaling components. Data points indicate mean log_2FC of normalized gene expression relative to axenic control plants of the corresponding genotype. Error bars indicate propagated standard error. Data from one experiment with n=1 replicates comprising 18 pooled plants per condition (measured in duplicate), sampled nine days post inoculation.

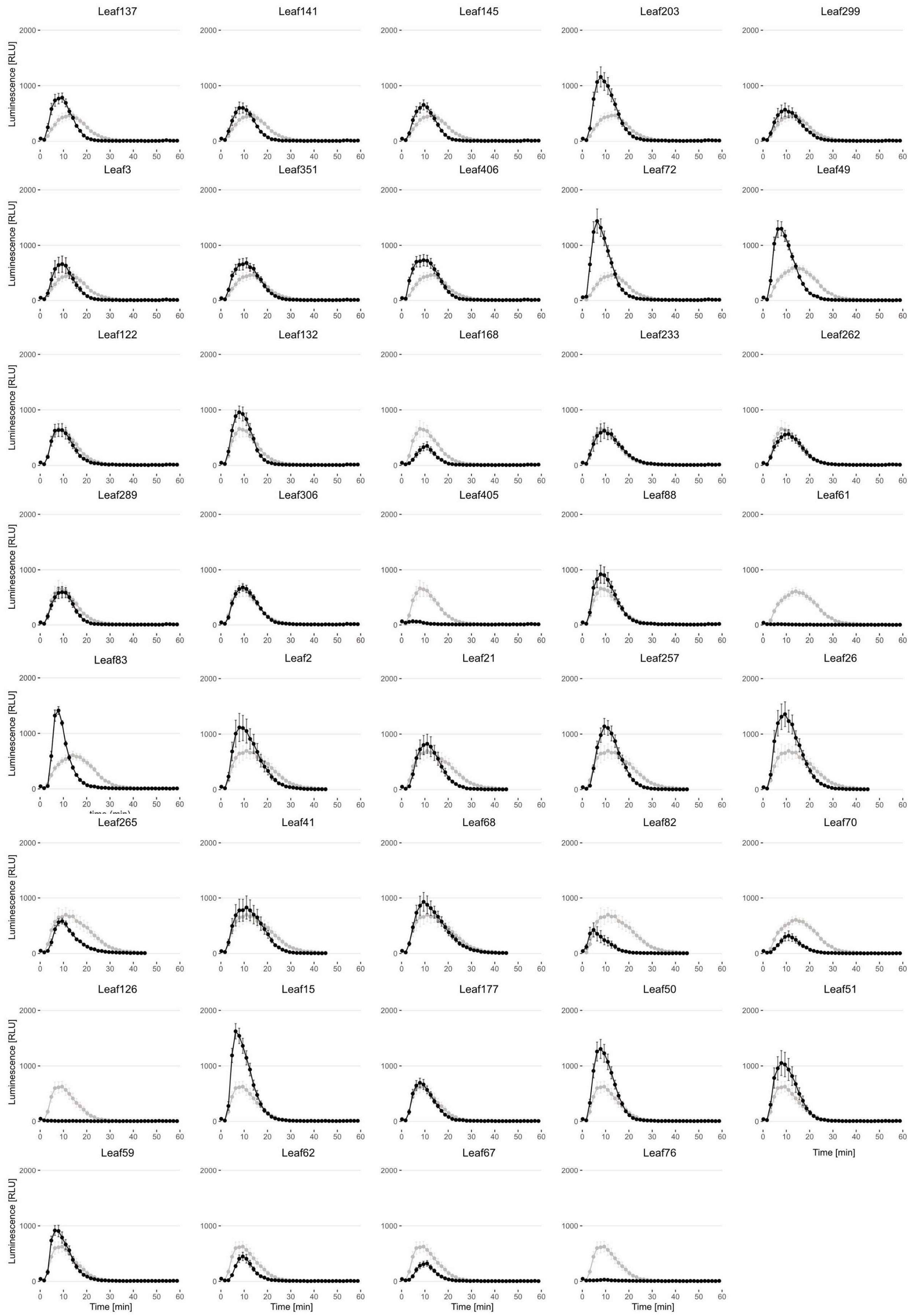

Suppl. Figure 7: Interference of bacterial extracts with flg22-mediated ROS burst in leaves. Curves show ROS accumulation in leaf discs upon treatment with 10 nM flg22 (grey) or 10 nM flg22 mixed with boiled culture extract (black) derived from 39 leaf microbiota strains (indicated on top of each plot), exhibiting various interference patterns. Extracts were made from bacterial suspensions normalized by density, resulting in a final OD_{600}-equivalent of 1 in the treatment solution. Points indicate mean, bars indicate standard error. Data from 8 leaf discs per condition.

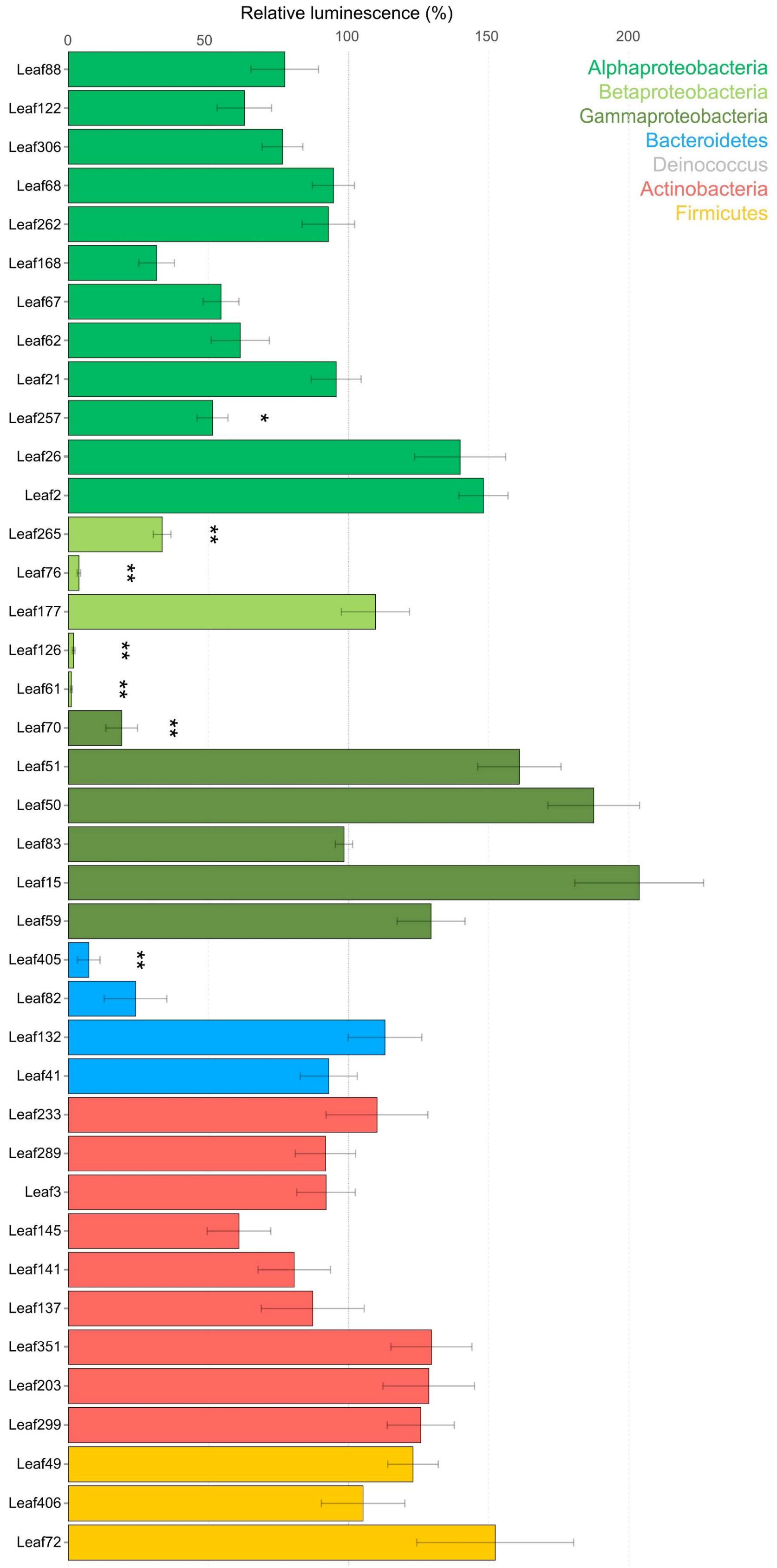

Suppl. Figure 8: ROS bursts elicited by flg22 in leaf-disks pretreated with leaf microbiota strains. Validation of data shown in Extended Data Fig. 9. Luminescence (x-axis) relative to 100 nM flg22 treatment in mock-pretreated leaf disks, indicated by vertical line at 100%. Strains (y-axis) ordered according to phylogeny, as highlighted by color. Columns indicate mean, error bars represent standard error. Statistical significance was assessed by a two-sided Wilcoxon test with Bonferroni correction relative to mock-pretreatment (**: $p \leq 0.01$, and *: $p \leq 0.05$). Data from n=1 experiments comprising 8 leaf discs per condition.

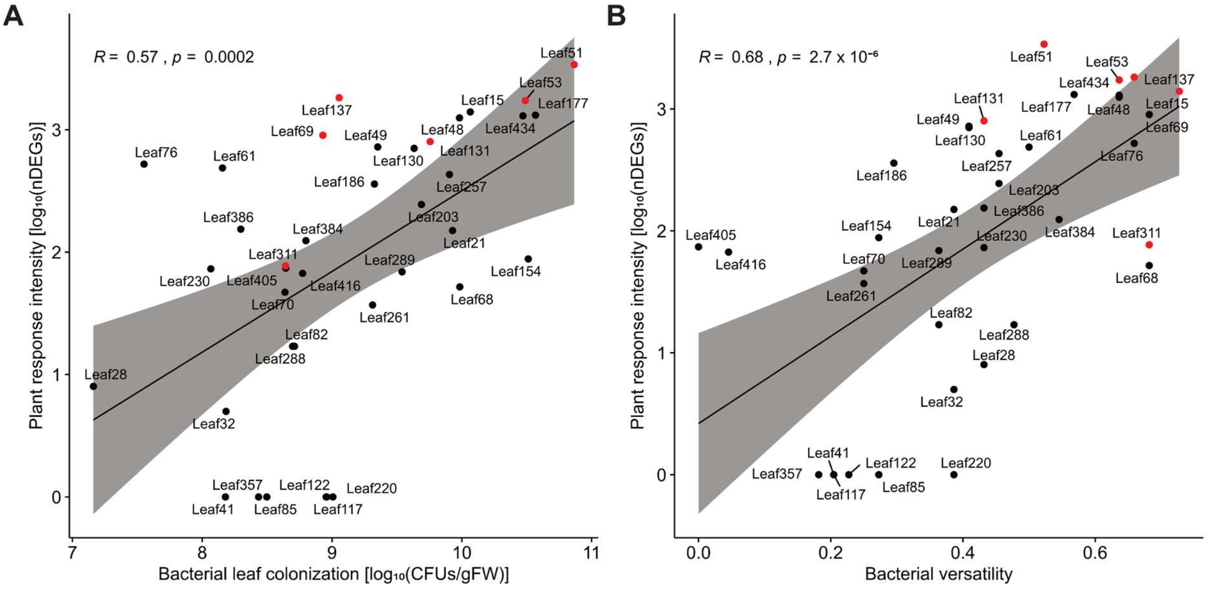

Suppl. Figure 9: Correlation analysis of bacterial population size and strain versatility in carbon source utilization with plant response intensity. (A) Correlation between bacterial abundance *in planta* (x-axis) and extent of plant transcriptional reprogramming (in number of differentially expressed plant genes (nDEGs), y-axis)in response to indicated leaf microbioa strains, as determined by Maier *et al.* (2021, ref.[21]). (B) Correlation between the extent of plant response (y-axis) to indicated strains (Maier *et al.* 2021) and their versatility in carbon source utilization (x-axis), as determined by Schäfer, Pacheco *et al.* (2023, ref.[83]). (A–B) *R* indicates Spearman's correlation coefficient, *p* inidcates P-value. Shaded area depicts 95 % confidence interval. Highlighted with a red dot are strains that were found to be enriched in mutant plants lacking GNSR components, as shown in Figure 1 and Extended Data Fig. 3.
