# Supplementary material for: Plant microbiota feedbacks through dose-responsive expression of general non-self response genes
Source: Nat Plants. 2024 Dec 3;11(1):74–89. doi: 10.1038/s41477-024-01856-z (PMC11757152; doi:10.1038/s41477-024-01856-z)
Supplement: Supplementary file 2 — Reporting Summary [file 41477_2024_1856_MOESM2_ESM.pdf]

nature portfolio

Corresponding author(s): Julia A. Vorholt

Last updated by author(s): Oct 12, 2024

Reporting Summary

Nature Portfolio wishes to improve the reproducibility of the work that we publish. This form provides structure for consistency and transparency in reporting. For further information on Nature Portfolio policies, see our Editorial Policies and the Editorial Policy Checklist.

Statistics

For all statistical analyses, confirm that the following items are present in the figure legend, table legend, main text, or Methods section.

n/a	Confirmed	
☐	☒	The exact sample size (*n*) for each experimental group/condition, given as a discrete number and unit of measurement
☐	☒	A statement on whether measurements were taken from distinct samples or whether the same sample was measured repeatedly
☐	☒	The statistical test(s) used AND whether they are one- or two-sided<br>*Only common tests should be described solely by name; describe more complex techniques in the Methods section.*
☒	☐	A description of all covariates tested
☐	☒	A description of any assumptions or corrections, such as tests of normality and adjustment for multiple comparisons
☐	☒	A full description of the statistical parameters including central tendency (e.g. means) or other basic estimates (e.g. regression coefficient) AND variation (e.g. standard deviation) or associated estimates of uncertainty (e.g. confidence intervals)
☐	☒	For null hypothesis testing, the test statistic (e.g. *F*, *t*, *r*) with confidence intervals, effect sizes, degrees of freedom and *P* value noted<br>*Give P values as exact values whenever suitable.*
☒	☐	For Bayesian analysis, information on the choice of priors and Markov chain Monte Carlo settings
☒	☐	For hierarchical and complex designs, identification of the appropriate level for tests and full reporting of outcomes
☐	☒	Estimates of effect sizes (e.g. Cohen's *d*, Pearson's *r*), indicating how they were calculated

Our web collection on statistics for biologists contains articles on many of the points above.

Software and code

Policy information about availability of computer code

Data collection	RNA sequencing data was obtained by Novogene (https://www.novogene.com) using an Illumina NovaSeq 6000. DNA sequencing data was obtained using an Illumina MiSeq. RT-qPCR data was obtained using an Applied Biosystems QuantStudio 7 system with QuantStudio Real-Time PCR System Version 1.3.
Data analysis	Data analysis was performed using RStudio Server (2022.7.0.548, RStudio Team) running R version 4.2.1. RNA sequencing data was processed using BBTools v 38.18, Salmon 14 v 1.10.1, and DESeq2 v 1.37.4. Exact processing commands are described in the Methods in Microbiomics database (https://methods-in-microbiomics.readthedocs.io). DNA sequencing data was processed using USEARCH (which includes UPARSE) v.11.0.667-i86 linux64. Custom scripts to analyze and visualize data can be found at https://github.com/MicrobiologyETHZ/phylloR. Regulatory network analysis was performed using ISMARA (Balwierz et al., 2014).

For manuscripts utilizing custom algorithms or software that are central to the research but not yet described in published literature, software must be made available to editors and reviewers. We strongly encourage code deposition in a community repository (e.g. GitHub). See the Nature Portfolio guidelines for submitting code & software for further information.

Data

Policy information about availability of data

All manuscripts must include a data availability statement. This statement should provide the following information, where applicable:

- Accession codes, unique identifiers, or web links for publicly available datasets
- A description of any restrictions on data availability
- For clinical datasets or third party data, please ensure that the statement adheres to our policy

RNA sequencing data was deposited at the European Nucleotide Archive under accession number PRJEB67453 (ERP152478). DNA sequencing data was deposited at the European Nucleotide Archive under accession number PRJEB80640 (ERP164609). Processing commands for RNA sequencing data are detailed on the Methods in Microbiomics database (https://methods-in-microbiomics.readthedocs.io).

Research involving human participants, their data, or biological material

Policy information about studies with human participants or human data. See also policy information about sex, gender (identity/presentation), and sexual orientation and race, ethnicity and racism.

Reporting on sex and gender	Not applicable.
Reporting on race, ethnicity, or other socially relevant groupings	Not applicable.
Population characteristics	Not applicable.
Recruitment	Not applicable.
Ethics oversight	Not applicable.

Note that full information on the approval of the study protocol must also be provided in the manuscript.

Field-specific reporting

Please select the one below that is the best fit for your research. If you are not sure, read the appropriate sections before making your selection.

☒ Life sciences ☐ Behavioural & social sciences ☐ Ecological, evolutionary & environmental sciences

For a reference copy of the document with all sections, see nature.com/documents/nr-reporting-summary-flat.pdf

Life sciences study design

All studies must disclose on these points even when the disclosure is negative.

Sample size	Sample sizes were based on similar experiments in previous studies (such as Maier et al., 2021). All experiments included a positive and/or negative control.
Data exclusions	All data that fulfilled quality control criteria were included in the study. E.g., low quality reads obtained from RNA sequencing were excluded or dead plants were not harvested.
Replication	Reproducibility of data was assured by including known conditions across experiments (e.g., inoculation of plants with Leaf137), comparison of data with previous work (e.g., Maier et al. 2021) or similar experiments, and/or including biologically independent replicates where possible (e.g., five replicate RNA sequencing experiments).
Randomization	Conditions were distributed randomly within experiments. E.g., agar-grown plants inoculated with a certain strain were distributed across three 24-well plates and paired with different conditions on each plate. This ensured randomization of samples during RNA/DNA extraction and subsequent measurements.
Blinding	Investigators were blinded to treatment groups during plant inoculation, harvest, sample processing, and measurement of samples.

Reporting for specific materials, systems and methods

We require information from authors about some types of materials, experimental systems and methods used in many studies. Here, indicate whether each material, system or method listed is relevant to your study. If you are not sure if a list item applies to your research, read the appropriate section before selecting a response.

Materials & experimental systems

n/a	Involved in the study
☒	☐ Antibodies
☒	☐ Eukaryotic cell lines
☒	☐ Palaeontology and archaeology
☒	☐ Animals and other organisms
☒	☐ Clinical data
☒	☐ Dual use research of concern
☐	☒ Plants

Methods

n/a	Involved in the study
☒	☐ ChIP-seq
☒	☐ Flow cytometry
☒	☐ MRI-based neuroimaging

Plants

Seed stocks	pCYP71A12::GUS, Cara Haney. jar1 ein2 npr1, rbohD rbohF, fls2, efr, cerk1, fls2 efr, fls2 efr cerk1 (fec), bak1 bkk1, bak1 bkk1 cerk1 (bbc), Cyril Zipfel. cyp71A12 cyp71A13, Erich Glawischnig. exo70B1 exo70B2, Marco Trujillo. prx71, NASC (N655911); gnsr1, NASC (N657631); gstf6, NASC (N672582); chx16, NASC (N681397); crk14, NASC (N686933); crk6, NASC (N695328); igmt3, NASC (N829786); mlo12, NASC (N872203). min7, min7 fls2 efr cerk1 (mfec), min7 bak1 bkk1 cerk1 (mbbc), Sheng Yang He.
Novel plant genotypes	Not applicable.
Authentication	All plant lines were subjected to genotyping prior to first amplification of seed stocks.
